# The anti-glioblastoma effect of cold atmospheric plasma treatment: physical pathway v.s. chemical pathway

**DOI:** 10.1038/s41598-020-68585-z

**Published:** 2020-07-16

**Authors:** Dayun Yan, Qihui Wang, Alisa Malyavko, Denis B. Zolotukhin, Manish Adhikari, Jonathan H. Sherman, Michael Keidar

**Affiliations:** 1 Department of Mechanical and Aerospace Engineering, George Washington University, Washington, DC 20052 USA; 2 School of Medicine and Health Science, George Washington University, Washington, DC 20052 USA; 3 Neurosurgery, School of Medicine and Health Science, George Washington University, Washington, DC 20052 USA

**Keywords:** Biomedical engineering, Mechanical engineering

## Abstract

Cold atmospheric plasma (CAP), a near room temperature ionized gas, has shown potential application in many branches of medicine, particularly in cancer treatment. In previous studies, the biological effect of CAP on cancer cells and other mammalian cells has been based solely on the chemical factors in CAP, particularly the reactive species. Therefore, plasma medicine has been regarded as a reactive species-based medicine, and the physical factors in CAP such as the thermal effect, ultraviolet irradiation, and electromagnetic effect have been regarded as ignorable factors. In this study, we investigated the effect of a physical CAP treatment on glioblastoma cells. For the first time, we demonstrated that the physical factors in CAP could reinstate the positive selectivity on CAP-treated astrocytes. The positive selectivity was a result of necrosis, a new cell death in glioblastoma cells characterized by the leak of bulk water from the cell membrane. The physically-based CAP treatment overcomed a large limitation of the traditional chemically based CAP treatment, which had complete dependence on the sensitivity of cells to reactive species. The physically-based CAP treatment is a potential non-invasive anti-tumor tool, which may have wide application for tumors located in deeper tissues.

## Introduction

Glioblastoma multiforme (GBM) is characterized as a highly invasive, aggressive brain tumor^1^. Individuals with GBM face a poor prognosis, with few surviving past the 2-year mark^1,2^. A combination of chemotherapy, surgical resection, and radiotherapy is the gold standard for glioblastoma therapy, however, each component has its own drawbacks^1,3,4^. Glioblastoma tumors generally originate deep in the brain and a new treatment option, particularly a non-invasive method, is needed to enhance the anti-cancer efficacy and decrease damage to normal tissues.

CAP is a cocktail containing different reactive oxygen species (ROS), reactive nitrogen species (RNS), other charged particles, neutral particles, and electrons as well as physical factors, such as thermal effect, ultraviolet (UV), and electromagnetic (EM) waves^5–7^. CAP has wide application in many areas, ranging from plasma chemistry, surface modification, decomposition of gaseous pollutants, medical sterilization, and microbial decontamination^8–12^. CAP also shows a wide application in cancer treatment^13–16^. CAP treatment has demonstrated strong and selective anti-cancer capacity in many cancer cell lines, including breast cancer, colorectal cancer, cervical cancer, skin cancer, and brain cancer^15^. CAP also effectively inhibits the growth of subcutaneous xenograft tumors as well as melanoma by a transdermal treatment above the skin of the tumor site^17^. In addition, some recent clinical trials have started to show the promising anti-tumor effect of CAP^18,19^.

To date, all reported anti-cancer effects of CAP treatment, both in vitro and in vivo have generally been regarded as the cellular responses to the chemical factors, particularly the reactive species^20–22^. Experiments using CAP-activated medium further support this conclusion^23–27^. H_2_O_2_ has been regarded as a key player resulting in plasma medicine being referred to as H_2_O_2_-medicine, but is also denoted as NO_2_^-^—medicine and other reactive species-based medicine in some cases^27–29^.

Similarly, the selective anti-cancer effect of CAP treatment is also regarded as the selective cellular response to the CAP-generated reactive species particularly H_2_O_2_^30^. When normal cells are more sensitive to the reactive species than the counterpart cancer cells, CAP treatment will only have negative selectivity. Therefore, conventional plasma medicine largely relies on reactive species, but at the same time, is naturally limited by the biological effect of reactive species.

To date, nearly all these studies have ignored the potential role of physical factors in the CAP cancer treatment. This is mainly due to the lack of clear evidence of the anti-cancer effect of the physical factors in CAP. Conventionally, when CAP treatment is performed, cancer cells are always covered by a thin layer of cell culture medium^31^. This layer of medium facilitates the solvation of short-lived reactive species in the gas phase and the formation of the long-lived reactive species in the liquid phase which act on the cells^32^. Recently, we demonstrated that even a thin layer of medium could block the physical effect of CAP on melanoma cells. This may be the reason behind the lack of investigation into the physical factors of CAP over the past couple of years^33^. The physical factors, mainly the EM emission from CAP, cause a new cell death in melanoma cell line B16F10. This new cell death results in a much stronger growth inhibition on the cancer cells compared with conventional chemically-based CAP treatment^33^.

In this study, we demonstrated the anti-glioblastoma effect of CAP treatment based on the physical factors of the CAP jet. Our experimental design blocked all potential chemical factors from interacting with the cells. The physical factors affected the cells in a non-invasive method through a physical barrier with a thickness of more than 1 mm. Compared with the traditional chemically-based CAP treatment, the physically-based CAP treatment not only showed stronger anti-glioblastoma effect, but also largely improved the side effect of chemical reactive species on human astrocyte cell line, hTERT/E6/E7.

## Methods and materials

### CAP jet device, chemical/physical treatment

**Figure 1:**
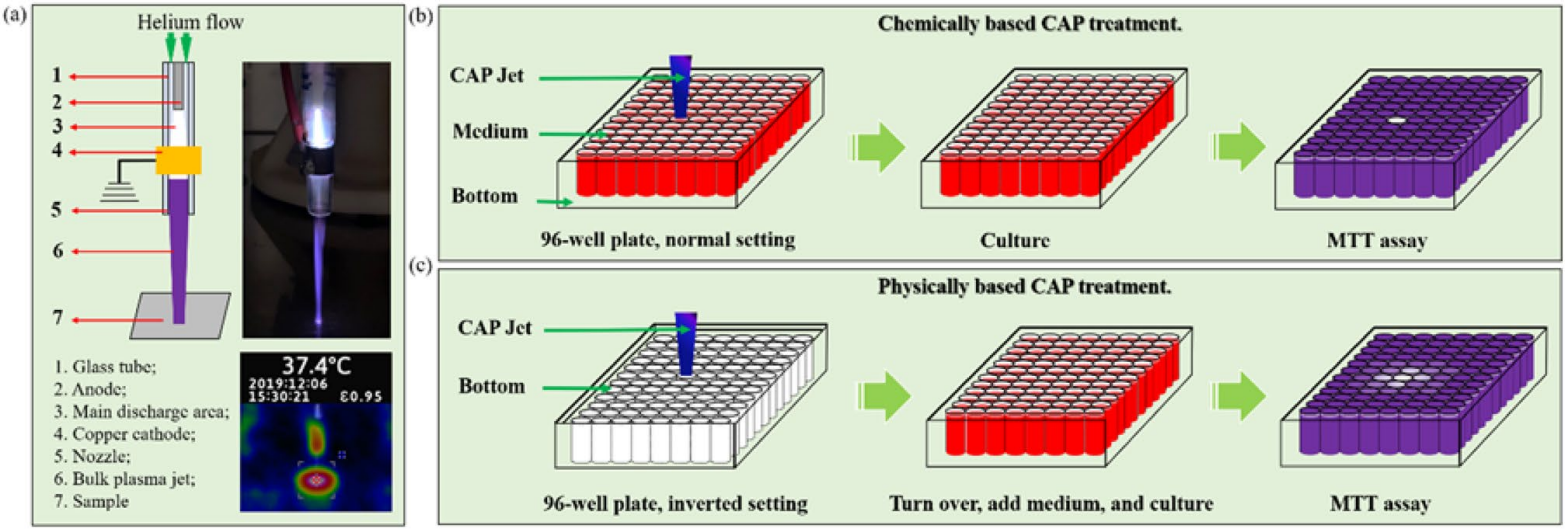
CAP jet and CAP treatment. (**a**) The CAP schematic illustration and photo of CAP jet. The discharge occurred between a coaxial stainless anode and another copper ring grounded cathode. Helium as the carrying gas take the ionized gas out the nozzle with a flow rate around 1.5 lpm. The diameter of the glass nozzle was 4.5 mm. An infrared photo (FLUKE Visual IR Thermometer) was shown in bottom panel. In the infrared photo, the CAP jet treated the bottom of a 35-mm dish. The temperature reflected the temperature of the dish’s center. (**b**) The schematic illustration of the chemically based CAP treatment. (**c**) The schematic illustration of the physically based CAP treatment. The cases of 12-well plates did not show at here.

Two CAP devices, based on different discharge ways, are widely used in plasma medicine^13,34^. One is based on the direct discharge, such as dielectric barrier discharge (DBD). Another one is based on the indirect discharge, such as CAP jet. In this study, we used a CAP jet as the CAP source. As show in Fig. 1a, the non-equilibrium discharge was trigged by an alternative voltage (3.16 kV, peak value) between two electrodes in the helium with a 99.995% purity (Roberts Oxygen, grade 4.5, size 300). The ionized gas flowed away from the main discharge arc area and enter the air containing N_2_ and O_2_ and finally form the violet jet containing plenty of reactive species and neutral particles. The temperature of the CAP-treated surface was less than 40 °C after a treatment lasting 8 min (Fig. 1a).

We adopted two treatment strategies. One was the traditional chemically based CAP treatment (Fig. 1b). The CAP jet directly touched the medium covering the cells. In this case, plenty of long-lived reactive species were formed in the medium and further affect the cells’ fate. The physical factors were blocked by this layer of medium^33^. This method was also the dominant method to perform direct CAP treatment in most kinds of literatures. Another strategy was based on the novel strategy we proposed in the recent publication, which was the easiest method to completely block all touch between the chemical factors in CAP and the cells but maximize the physical effect of bulk CAP on cells through a physical barrier (Fig. 1c)^33^. Through the treatment on the bottom of a multi-well plate such as 96-well plate and 12-well plate or a cell culture dish such as 35-mm dish and 60-mm dish, the chemical factors particularly the reactive species would be blocked because they could not penetrate a thick polystyrene material^33^.

### Cell culture

In this study, a glioblastoma cell line U87MG and a normal human astrocyte cell line hTERT/E6/E7 were donated by Prof. Sherman’s lab at the George Washington University. U87MG cells and hTERT/E6/E7 cells were cultured in a complete medium composed of Dulbecco’s Modified Eagle Media (DMEM, Life Technologies) supplemented with 10% (v/v) fetal bovine serum (FBS, GE Healthcare, SH30396) and 1% (v/v) antibiotic solution (penicillin and streptomycin, Life Technologies) under the standard cell culture conditions (a humidified, 37℃, 5% CO_2_ environment). The typical microscopic images of cells were shown in Supplementary Fig. S1.

### Cell viability assay and 2D cell viability maps

**Figure 2:**
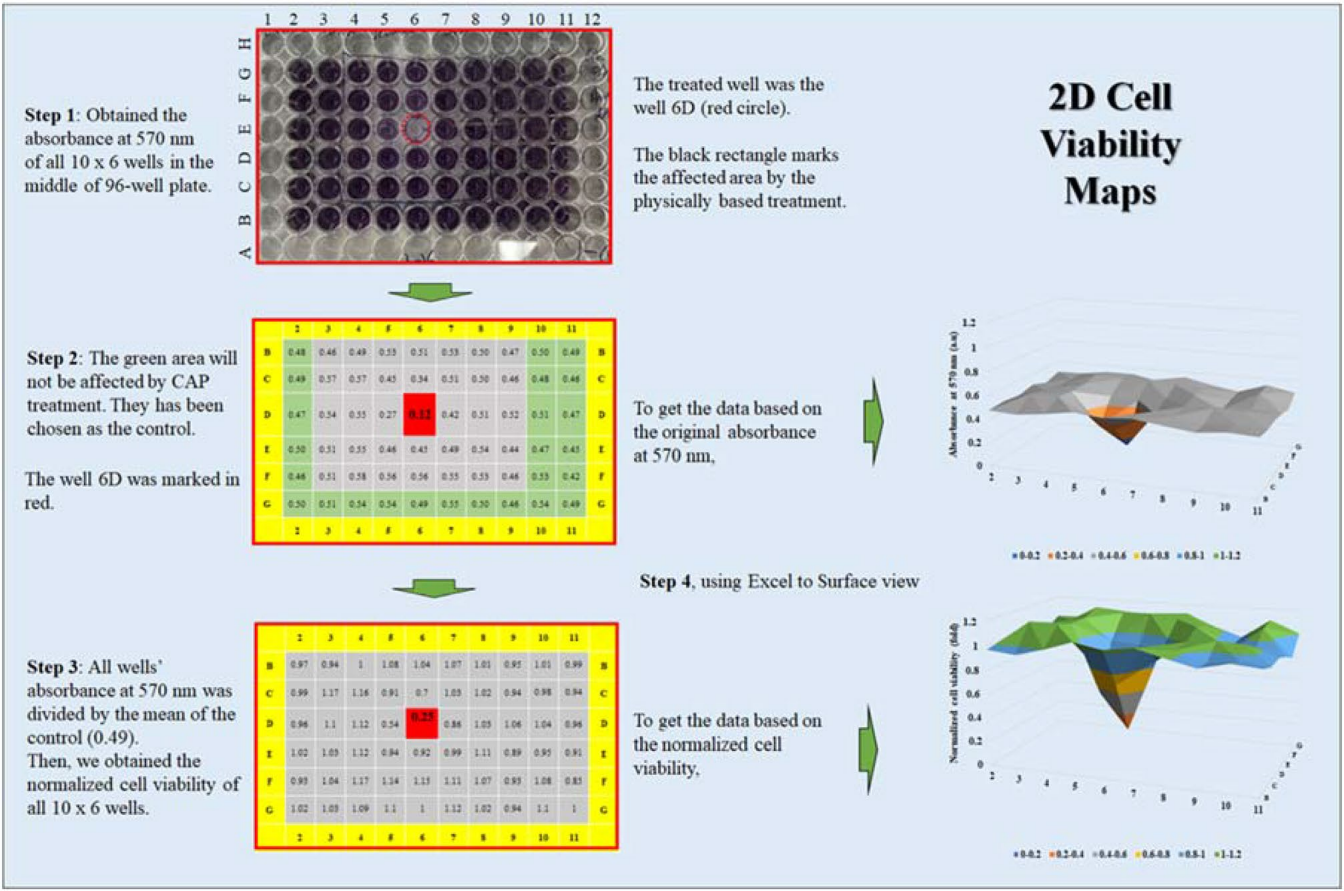
The protocols to make a 2D cell viability assay map based on a 96-well plate. The photo of a 96-well plate after the MTT assay was used here as an example. To get the map reflecting the original absorbance at 570 nm, the original data were used. To obtain the map reflecting the normalized data, the original data would be divided by the mean value of the control group. The data used to draw the 2D maps was the mean of the repeated tests. Each well’s data were based on the mean value of the corresponding well’s data in different repeats. The standard deviation (s.d.) of each well was also obtained, if needed, they could be shown in 2D maps based on s.d.

MTT (Thiazolyl Blue Tetrazolium Bromide) assay (Sigma-Aldrich, M2128) was used to perform the cell viability assay. MTT assay was performed in two steps following the standard protocols. The absorbance of the final solution at 570 nm was measured using a microplate reader (Synergy H1 Microplate Reader, Hybrid Technology). For the 12-well plate, the relative cell viability was obtained by the division between the experimental group and the control group. For 96-well plate, the relative cell viability was demonstrated via a new expression way, 2D cell viability map (Fig. 2), which has been first introduced in the previous study^33^. 2D cell viability maps reveal the viability of all 10 × 6 wells in the middle of the 96-well plate. The use of 2D cell viability maps was based on the previous observation that the physical effect on cells will affect a much larger area compared with the diameter of a single well of 96-well plate 33. 2D cell viability maps could reflect the spatial distribution of the biological effect of CAP on the cells.

### The chemically-based CAP treatment

The chemically-based CAP treatment was performed in the way shown in Fig. 1b. Specifically, the medium used in the overnight culture was removed first. For 96-well plate and 12-well plate, the protocols were similar. 1,000 μL/well and 100 μL/well of fresh DMEM was added to cover cells in 12-well plate and 96-well plate, respectively. For the chemically based treatment, the impact of CAP treatment would only be limited to the well touching the CAP jet^33^. Thus, the neighboring wells could be used as the experimental groups. For 96-well plates, however, only a single well the well 6D was treated by the CAP jet. After the treatment, the cells were further cultured for 2 days or 3 days under the standard culture conditions, followed by a cell viability assay.

### The physically-based CAP treatment

**Figure 3:**
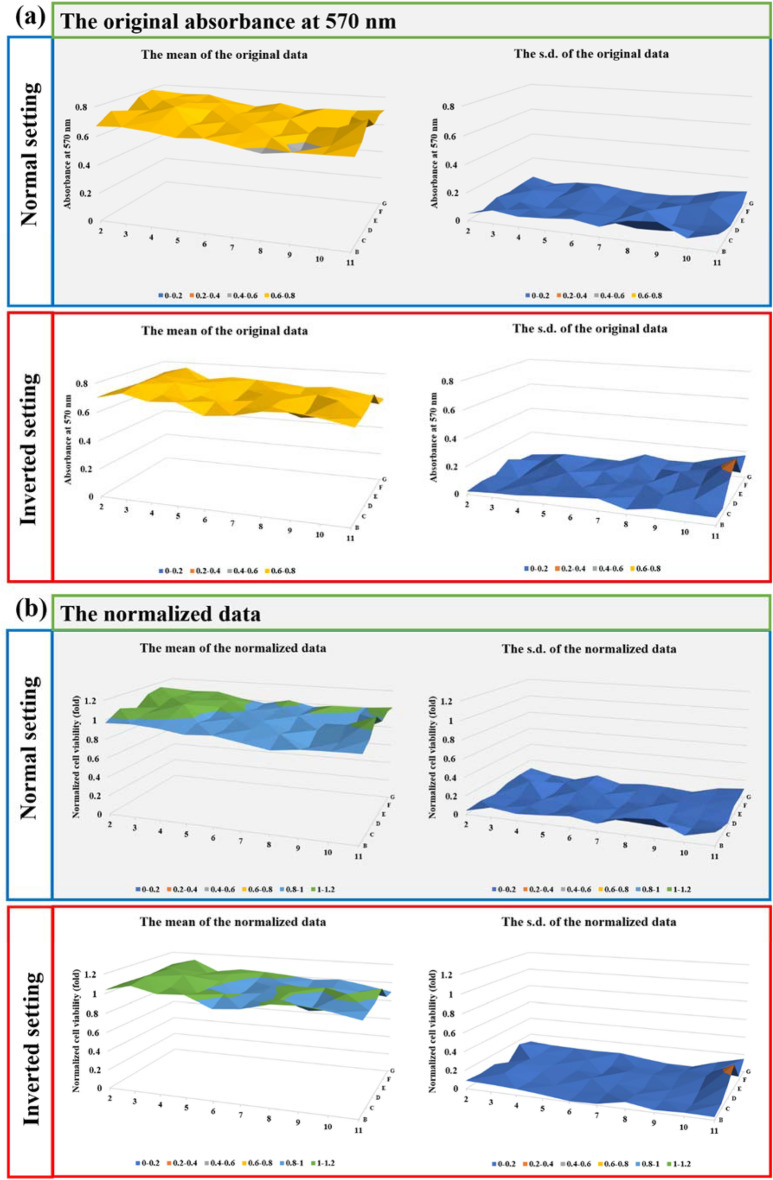
Just the inverted setting of the 96-well plate did not affect the cell growth (U87MG). (**a**) The original data based on the absorbance at 570 nm. (**b**) The normalized data. These 2D cell viability maps tend to show the cell viability after the normal setting and the inverted setting of 20 min. During the inverted setting, the bulk medium was moved away. Both the 2D maps based on the mean value of the repeated experiments and the 2D maps based on the s.d. of the repeated experiments were shown here. Results are presented as the mean ± s.d. of the experiments repeated 2 times.

The physically-based CAP treatment was performed on the bottom of inverted multi-well plates or cell culture dishes (Fig. 1c). Here, we just used a 12-well plate and 96-well plate as examples to specifically introduce the protocols. First, the medium in a 96-well plate was removed after a 24 h of culture. Despite there was no bulk medium left to cover the cells during the treatment, a very thin layer of the medium might still be left around the cells due to the surface tension of water. The cell viability of the control after such an inverted setting would not show decrease during the treatment time as long as 20 min (Fig. 3). When CAP jet treated cells, the diameter of the jet was much smaller than the diameter of a single well on a 12-well plate. Thus, the cells in the neighbor wells won't be affected by the treatment in another cell. For 96-well plates, however, only a single well the well 6D was treated by the CAP jet. After the treatment, 100 μL/well of DMEM and 1,000 μL/well of DMEM was added to culture the cells in the whole middle 10 × 6 wells on a 96-well plate and each well on 12-well plates, respectively. The cells were further cultured for 2 days or 3 days under the standard culture conditions, followed by a cell viability assay.

## Results

### The growth inhibition of U87MG cells by physically-based treatment

**Figure 4:**
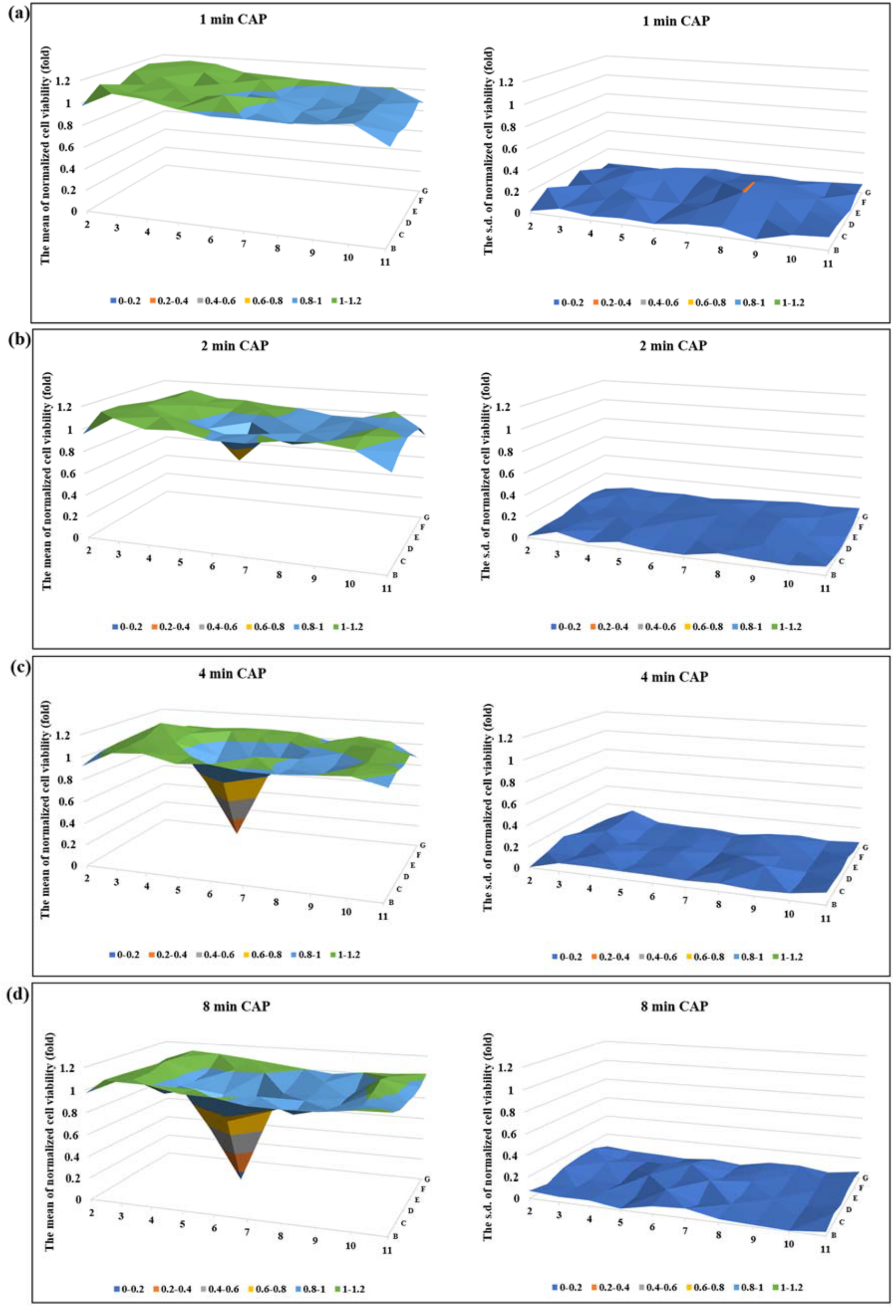
The physically-based CAP treatment effectively inhibited the growth of glioblastoma cells. The CAP treatment was performed for 1 min (**a**), 2 min (**b**), 4 min (**c**), and 8 min (**d**). The treatment was performed on the bottom of the 96-well plate, targeting the well 6D. For each case, 100 μL/well of U87MG (12 × 10^4^ cell/mL) were seeded in 96-well plate and cultured for 7 h before the treatment. The cells were cultured for 3 days before the final MTT assay. The normalized cell viability was obtained by using the protocols shown in Fig. 2. In each case, the mean value and the s.d. of the normalized cell viability were presented in the left panel and the right panel, respectively. Results were presented as the mean ± s.d. of the experiments repeated for 4 times. The original data were shown in Supplementary Fig. S2.

Physically-based CAP treatment was performed on U87MG cells in 96-well plates. The 2D cell viability maps depicting growth inhibition following CAP treatment were shown in Fig. 4. Noticeable growth inhibition was observed when treatment time extended past 1 min. Longer treatment time not only led to more significant growth inhibition on the well the CAP jet directly interacted with, well 6D, but also resulted in increased growth inhibition of the area surrounding well 6D. For example, when the treatment time was 8 min, the U87MG cells at well 6D had a normalized cell viability of just 12.0% ± 9.6%. Nearly all the wells surrounding well 6D experienced reduced cell viability. Based on the original data shown in Supplementary Fig. S2, the normalized cell viability of well 6C, 7D, 7E, 6E, 5E, and 5D was 74.3% ± 12.0%, 79.1% ± 14.2%, 88.6% ± 5.4%, 74.2% ± 17.3%, 83.5% ± 13.9%, and 57.5% ± 12.5%, respectively. Observing an effect on an area much larger than the diameter of jet, the direct area the jet is touching, is a typical feature of physically-cased CAP treatment.

### Different treatment, different selectivity

**Figure 5:**
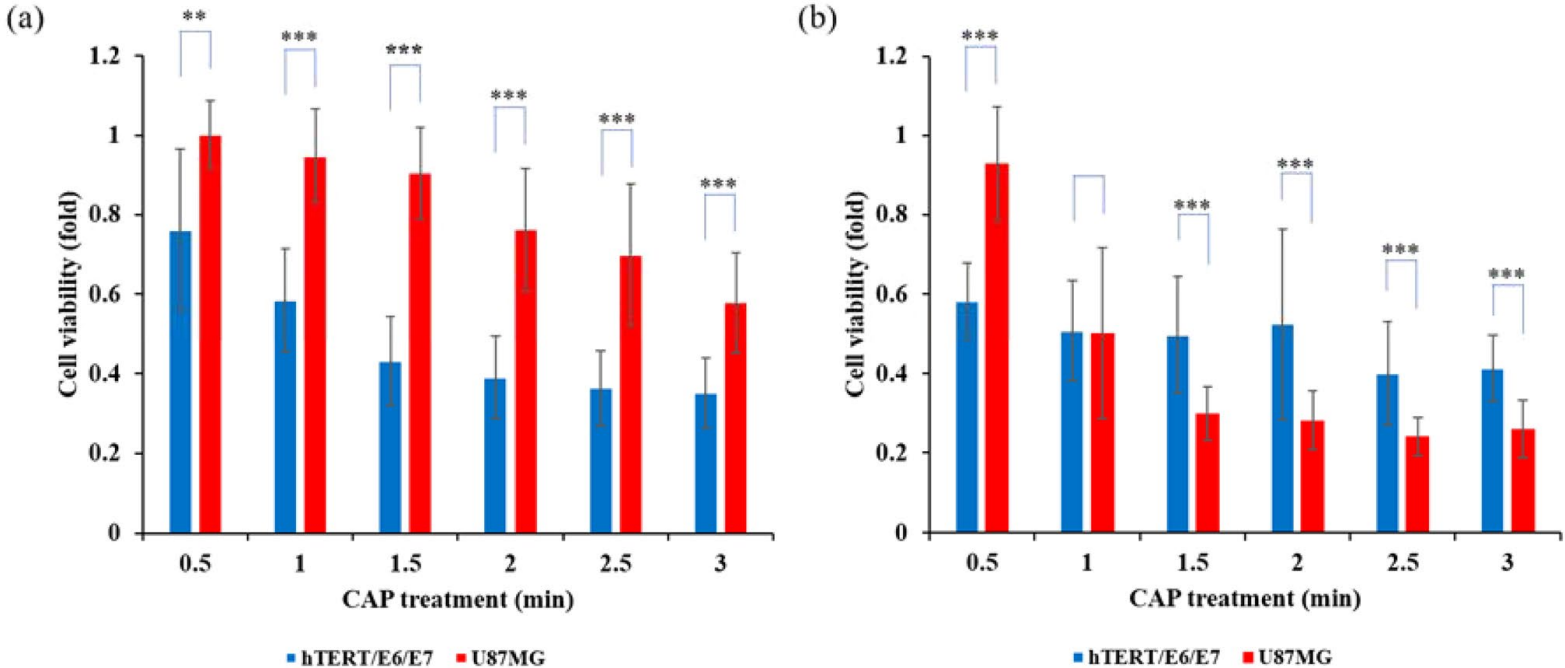
The negative and positive selectivity in both CAP treatment strategies. (**a**) The chemically-based CAP treatment. (**b**) The physically-based CAP treatment. For each case, 1 mL/well of U87MG or hTERT/E6/E7 cells (5 × 10^4^ cell/mL) were seeded in 12-well plates and cultured for 7 h before the treatment. The cells were cultured for 3 days before the final MTT assay. The normalized cell viability was obtained by the division between the experimental group and the control group. Results were presented as the mean ± s.d. of the experiments repeated 3 times. Student’s t-test was performed, and the significance was indicated as *p < 0.05, **p < 0.01, ***p < 0.005.

We compared the growth inhibition following the chemically-based CAP treatment on a glioblastoma cell line U87MG and an astrocyte cell line hTERT/E6/E7. Because the cells’ growth speeds were quite different, the two cell lines were cultured 7 h before treatment to allow for the densities of two cell lines to be approximately the same prior to CAP treatment. As shown in Fig. 5a, the normal cell line hTERT/E6/E7 was much more sensitive to the chemical CAP treatment than the glioblastoma cell line U87MG. We referred to such a cellular response as negative selectivity of CAP cancer treatment. This phenomenon of negative selectivity has been previously reported in other cell lines^30,35^. The negative selectivity was completely determined by the high sensitivity of this normal cell line to the CAP-originated reactive species. In contrast, when the physically-based CAP treatment was performed, the growth inhibition on U87MG cells was stronger than that on hTERT/E6/E7 cells, particularly when the treatment time was longer than 1 min (Fig. 5b). This was a typical positive selectivity. In other words, although hTERT/E6/E7 cells did show some growth inhibition after physically-based CAP treatment, they showed less sensitivity to the physical factors in CAP when compared to U87MG cells.

### The new cell death of U87MG cells is initially triggered by membrane bubbling

**Figure 6:**
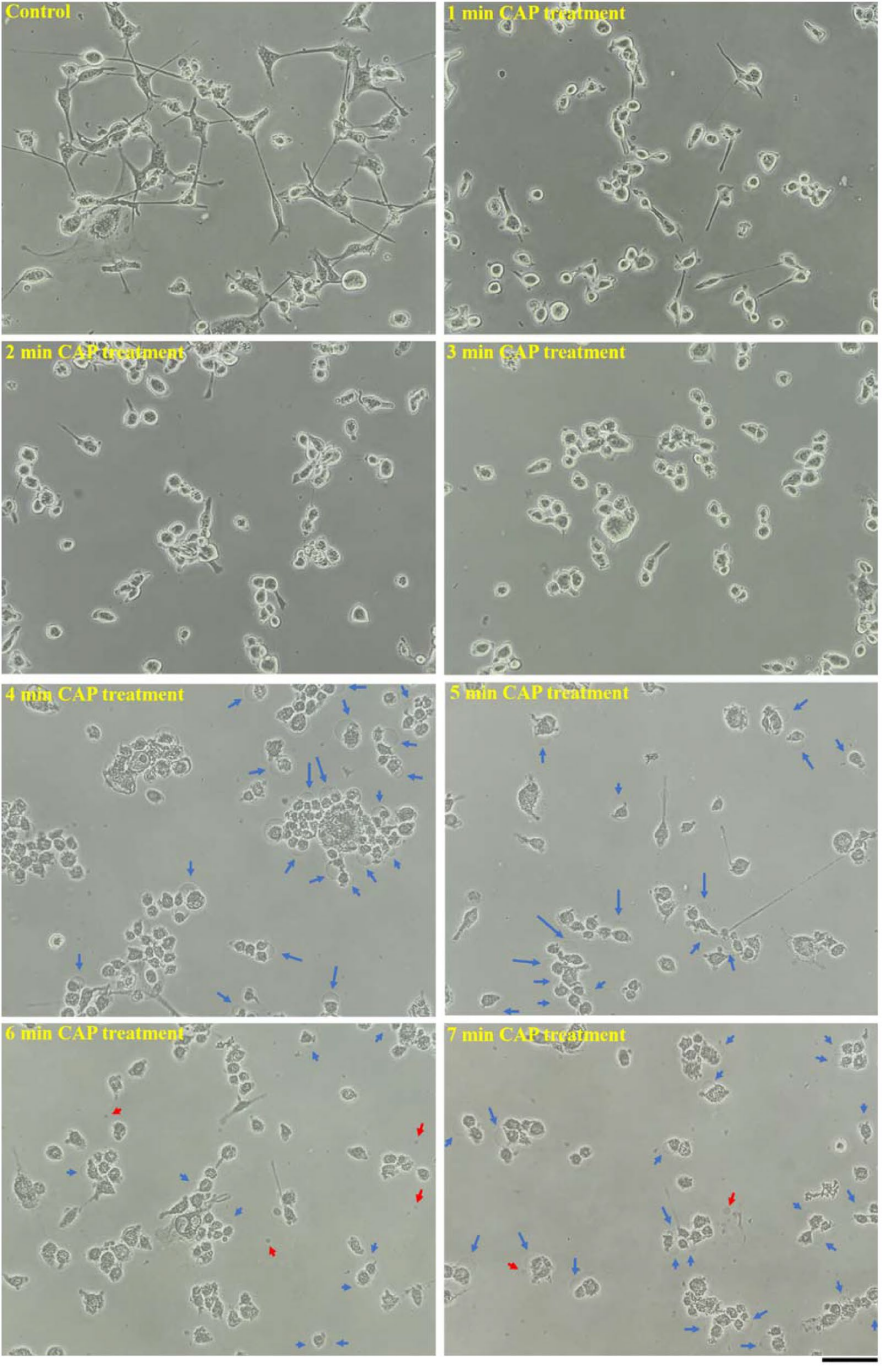
The morphological change of U87MG cells after the physically-based CAP treatment. For the control group, the medium was also renewed. No CAP treatment was performed before imaging. All photos were taken at 11 min after the treatment. The clear bubbles on the cellular membrane and the clear detached bubbles in the extracellular space were marked by blue arrows and red arrows, respectively. 1.5 mL of U87MG cells (7.5 × 10^4^ cell/mL) were seeded in 35 mm dishes and cultured for 24 h in the incubator. The scale bar was 100 μm (black). All photos were taken by using a Nikon TS100 inverted phase-contrast microscope.

To observe the cell death type caused by the physically based CAP treatment, microscopic observation was performed on the cells seeded in 35 mm and 60 mm cell culture dishes rather than multi-well plates because multi-well plates were not suitable for microscopic observation. Microscopic observation was done using a Nikon TS100 phase-contrast microscope. We first investigated the morphological change of U87MG cells after CAP treatment of different time spans to illustrate when specific morphological changes begin. As shown in Fig. 6, a clear morphological change on U87MG cells of drastic cytoplasm shrinkage could be seen when the treatment time was 1 min. When the treatment time was extended to 4 min, U87MG cells experienced further morphological change in the form of bubbling. Clear bubbles could be seen on the cellular membrane of many cells. When the treatment time continued to increase, more bubbles appeared in the extracellular space, which could have been due to the detachment of the bubbles from the cells.

**Figure 7:**
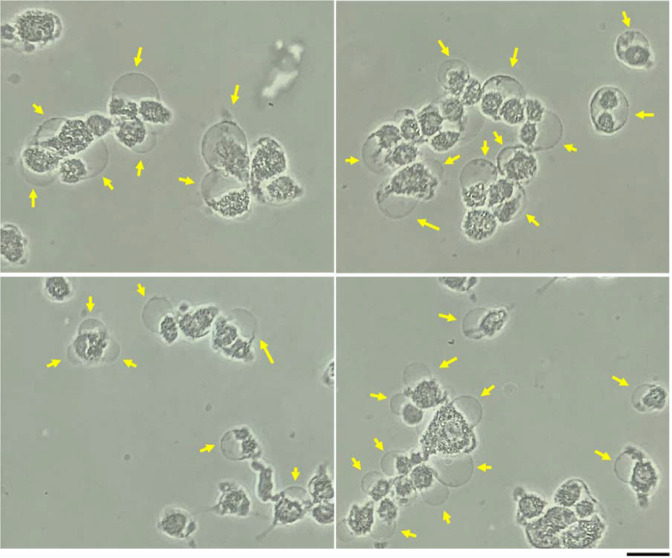
The typical photos of the bubbles on the CAP-treated U87MG cells. Here, we showed four photos of the typical bubbling. A schematic illustration of the bubbling on the cell is shown in the middle. The photos were taken at 11 min post a 4 min of CAP treatment. The clear bubbles were marked by yellow arrows. 1.5 mL of U87MG cells (7.5 × 10^4^ cell/mL) were seeded in 35 mm dishes and cultured for 24 h in the incubator. The scale bar was 50 μm (black). In each row, the photos were taken in situ. All photos were taken by using a Nikon TS100 inverted phase-contrast microscope.

Four photos of the membrane bubbling observed on CAP-treated U87MG cells were presented in Fig. 7. These photos were taken at 11 min after the treatment. These bubbles could be as large as U87MG cells, with some bubbles growing to twice the size of the cell’s cytoplasm. The transparent nature of these bubbles indicated that they might be composed of water or a solution without organelles. This speculation has been confirmed in our lab’s observation of melanoma cells post-CAP treatment using fluorescent imaging^33^. A plasma membrane might be main component to form the interface to distinguish the bubbles from medium.

**Figure 8:**
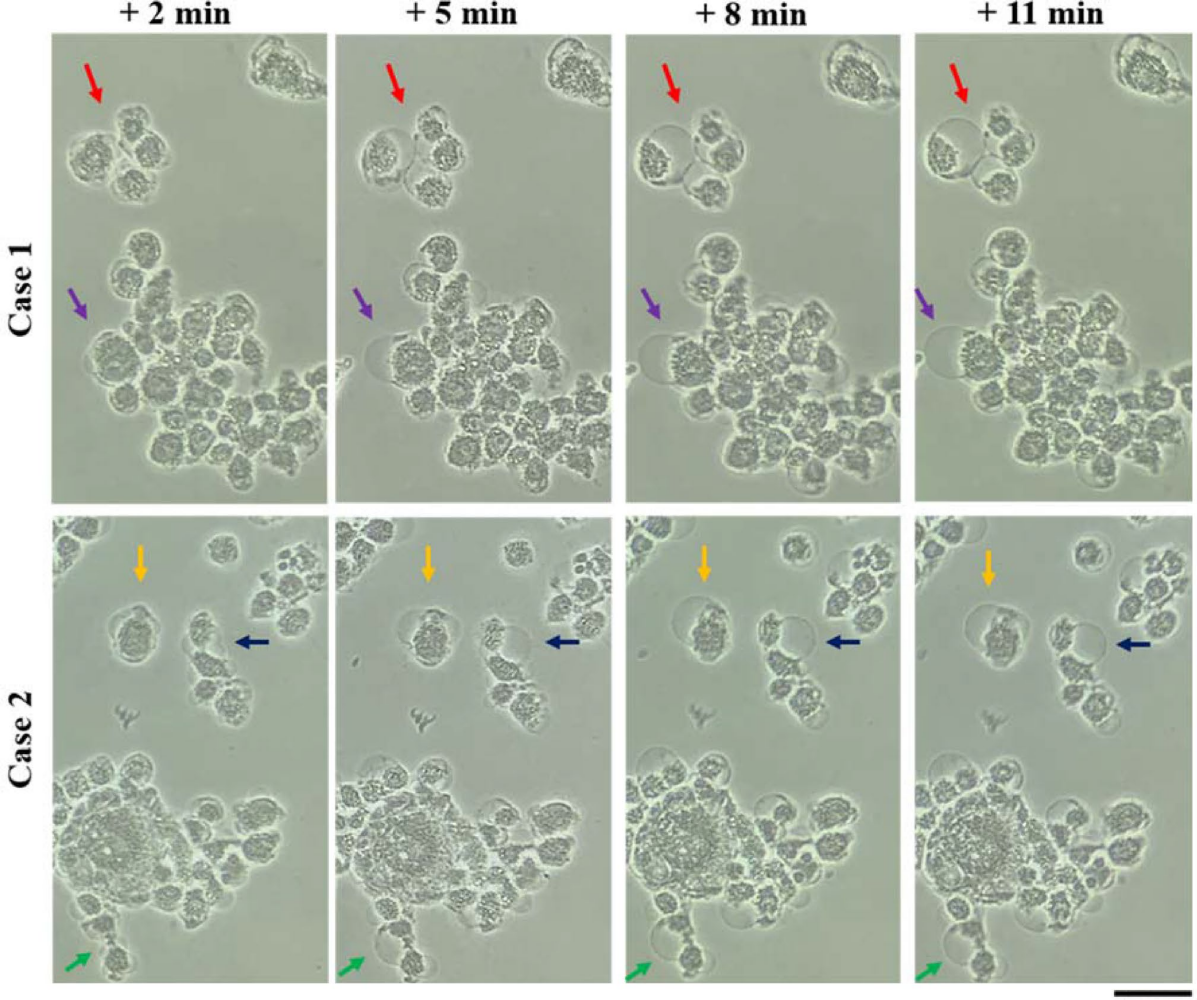
The growth of bubbles on the CAP-treated U87MG cells. Two cases were shown here. The photos were taken after 4 min of CAP treatment. The arrow with a specific color marked a specific bubble on the cytoplasm membrane. 1.5 mL of U87MG cells (7.5 × 10^4^ cell/mL) were seeded in 35 mm dishes and cultured for 24 h in the incubator. ‘ + x min’ meant the photo was taken at x min after the CAP treatment. The scale bar was 50 μm (black). In each row, the photos were taken in situ. All photos were taken by using a Nikon TS100 inverted phase-contrast microscope.

It is necessary to point out that the growth of the bubbles and the shrinkage of the cytoplasm did not happen simultaneously. As shown in Fig. 6, the cytoplasm shrinkage occurred immediately after a 1 min of CAP treatment. The growth of bubbles tentatively occurred after the third minute of treatment because the bubbles were visualized after 4 min of CAP treatment. We further captured the growth process (11 min) of several single bubbles on U87MG cells after 4 min of CAP treatment. As shown in Fig. 8, the growth of a single bubble generally ceased at the 8th minute post-CAP treatment. The same trend was observed in the melanoma cells discussed in our recent publication^33^. It is still unknown whether the growth of bubbles usually halts around 8 min in different cell lines. After the 8-min mark, the sizes of the bubbles did not change prior to their final detachment in the following hours. Due to the detachment, the bubbles were observed one day or two days after the treatment. Another noticeable feature was that the expansion of a bubble tends to be accompanied by the bubble’s color becoming lighter, possibly indicating the dilution of the components in the bubble.

After all of the bubbles detached from the cell membrane, the cell morphology continued to change over the following days until the end of the cell culture. This trend matched the trend reported in the CAP-treated melanoma cell line B16F10^33^. It is necessary to point out that the physically treated U87MG cells did not migrate, duplicate, or show any cellular activity. Therefore, they were presumed to be dead, which was different from a typical apoptotic process.

### The cellular changes on CAP-treated astrocyte cell line hTERT/E6/E7

**Figure 9:**
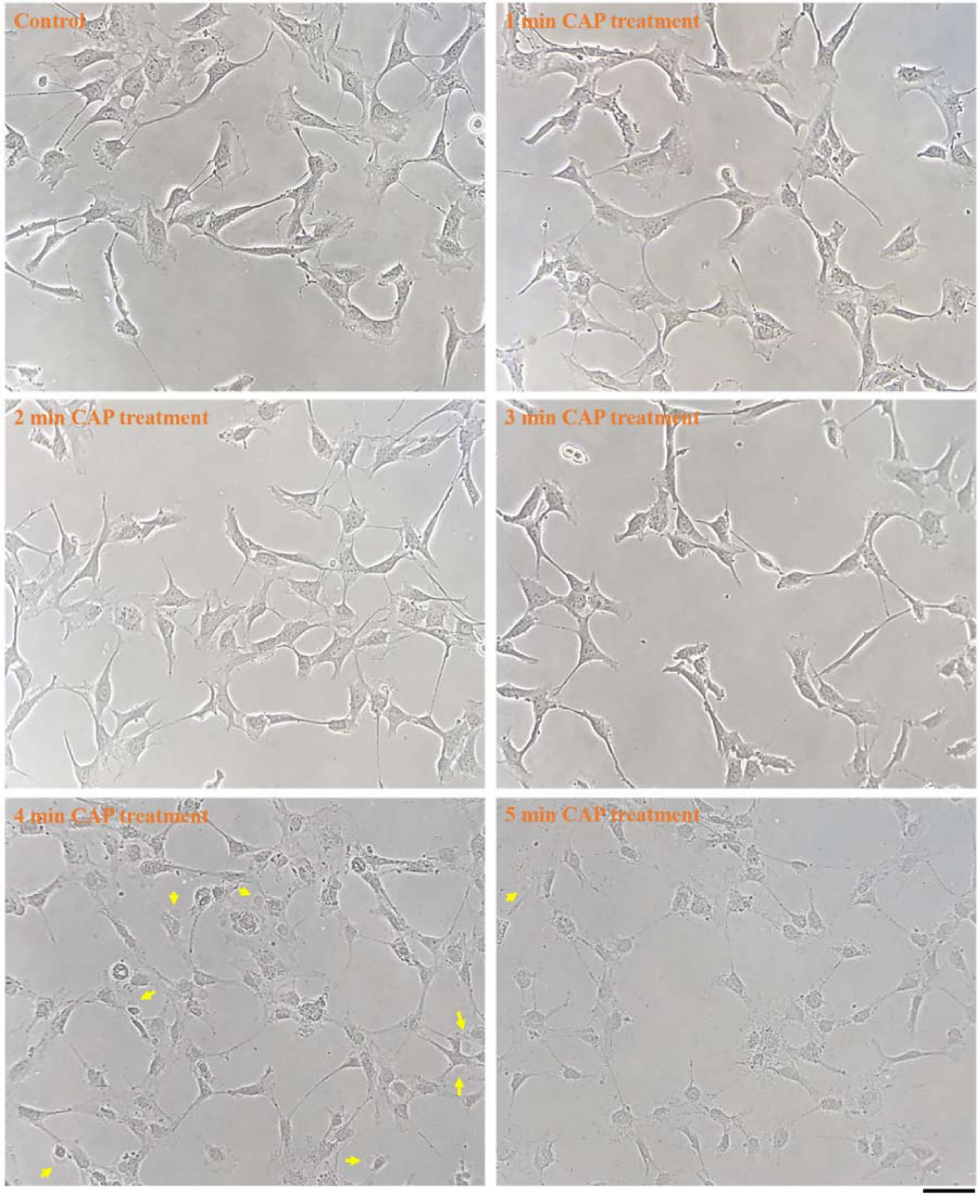
The morphological change of the physically-based CAP-treated hTERT/E6/E7 cells. For the control group, the medium was also renewed. No CAP treatment was performed before imaging. The photos were taken at 11 min after the CAP treatment. The clear bubbles on the cellular membrane were marked by yellow arrows. 2 mL of hTERT/E6/E7 cells (3 × 10^4^ cell/mL) were seeded in 35 mm dishes and cultured for 48 h in the incubator. The scale bar was 100 μm (black). All photos were taken by using a Nikon TS100 inverted phase-contrast microscope.

In contrast, a physical CAP treatment caused a less drastic cellular change of the normal astrocyte hTERT/E6/E7. A noticeable morphological change of the astrocyte cell line was not noticeable until the CAP treatment time was longer than 3 min (Fig. 9). When the treatment time increased to 4 min, some level of aggregation of the nucleus was noted but aggregation of the cytoplasm was not observed. The color of the cytoplasm was noticeably lighter, compared to the case with a shorter treatment time, and bubbles were only seen on a few single cells. Compared with U87MG cells, the bubble density on the normal cells hTERT/E6/E7 was insignificant. Even when the CAP treatment time was extended to 5 min, the typical features of the new cell death on U87MG cells were not observed on astrocyte hTERT/E6/E7. No apoptotic features were seen in the astrocyte cell line as well. The change seen in the astrocyte cell line was shown in Supplementary Fig. S3. The microscopic observation of the physical effect of CAP treatment on U87MG and hTERT/E6/E7 explained the positive selectivity of the physical CAP treatment.

### The bubbling may be due to physically-triggered intracellular pressure

The physically-triggered bubbling did not only appear during treatment of the bottom of the multi-well plate and dishes, but also appeared when CAP treatment directly touching the U87MG cells was performed. We have recently demonstrated that the medium layer covering the cells during in vitro experiments was the key factor in shifting the effect from chemical CAP factors to physical CAP factors^33^. In other words, the water layer or the medium layer blocked the impact of physical factors in CAP. In this study, we also found that the medium played a key role in the chemical to physical factors switch. In this section, we explored the bubbling mechanism based on the direct CAP treatment of U87MG cells without the coverage of medium.

**Figure 10:**
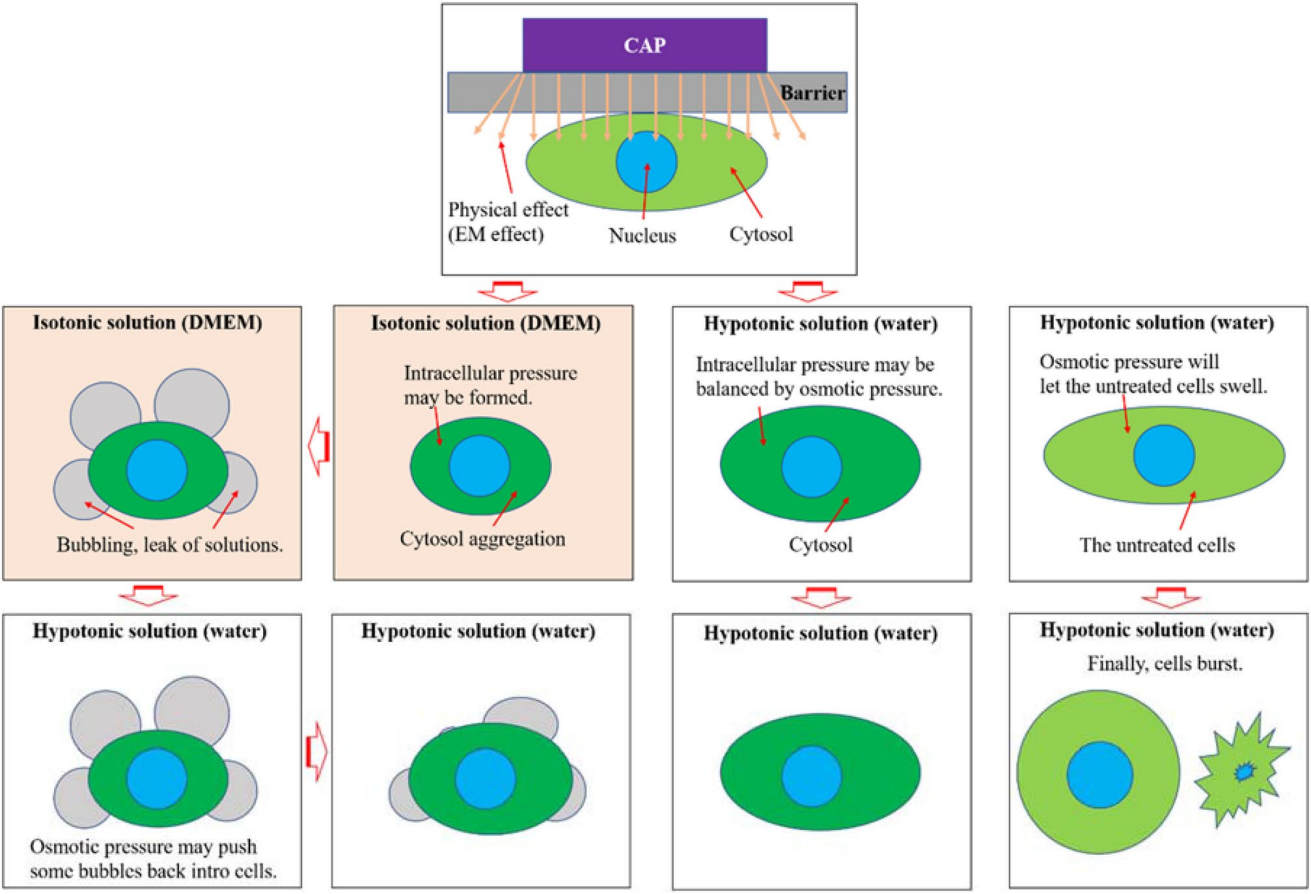
A schematic illustration for the role of osmotic pressure on the bubbling.

**Figure 11:**
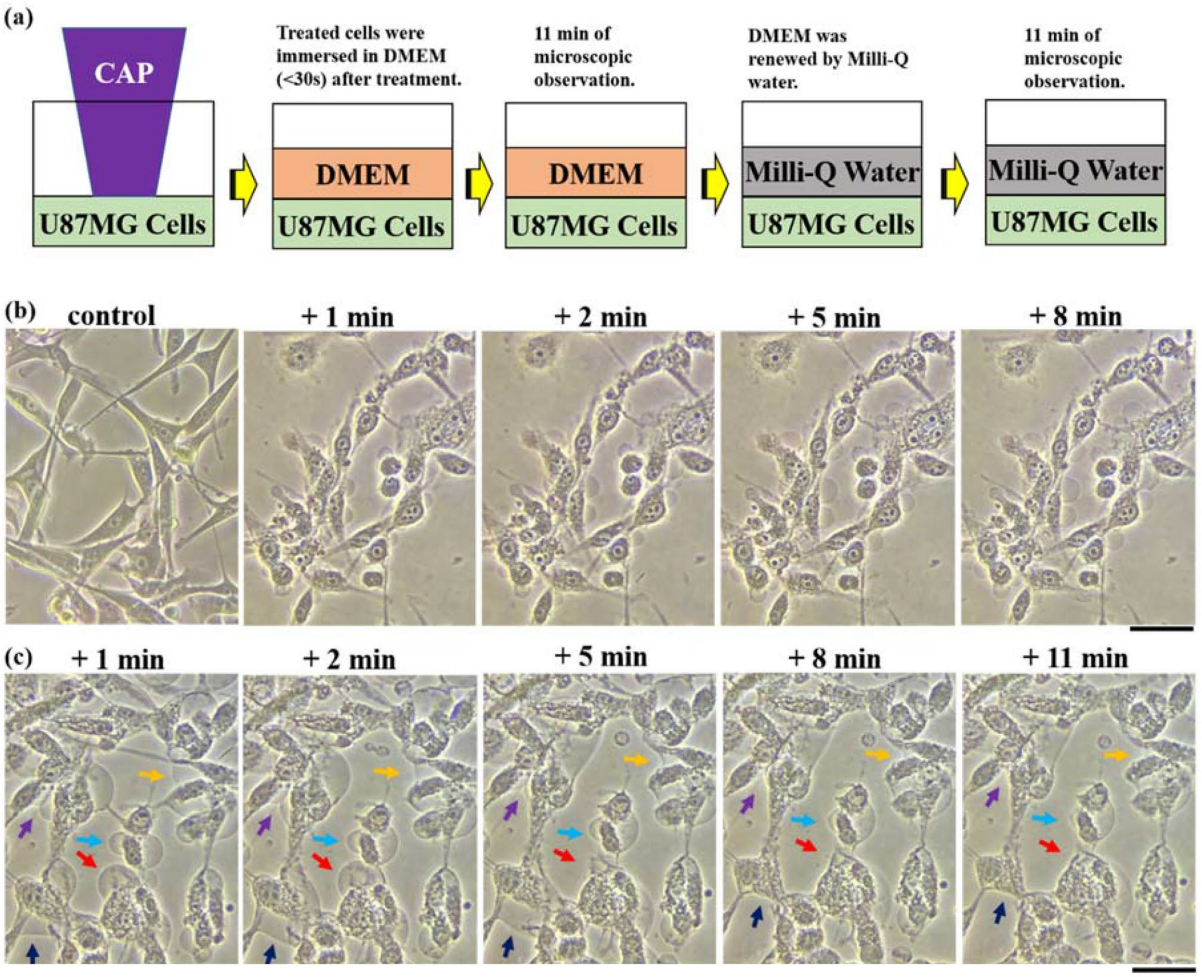
Some bubbles can be pressed back into U87MG cells in Milli-Q water. (**a**) A schematic illustration of the protocols. (**b**) The growth of bubbles in DMEM after the CAP treatment. (**c**) The cellular response when the cells with bubbles were immersed in Milli-Q water. 6 mL cell solution was cultured in a 60 mm dish with a density of 7.5 × 10^4^ cells/mL for one day before the treatment. For the control group, the medium was also renewed. No CAP treatment was performed before imaging. The bubbling on U87MG cells was first generated after a direct 2 min of CAP treatment. 8 min of the bubbling process was further recorded when the cells were immersed in DMEM. Afterward, DMEM was quickly (< 1 min) removed and renewed by 6 mL of Milli-Q water. The change of bubbles was recorded following this step. ‘ + x min’ means the photo was taken x min after the treatment or after cells were immersed in Milli-Q water. In the second row, specific bubbles were marked by specific colors. The scale bar was 50 μm (black). In each row, the photos after the treatment were taken in situ. All photos were taken by using a Nikon TS100 inverted phase-contrast microscope.

When cells, particularly mammalian cells such as cancer cells, are immersed in a hypotonic solution like deionized water, the influx of water into the cells through the cell membrane is a fundamental cellular response to equalize the osmotic pressure^36^. The U87MG cells without experiencing the CAP treatment would gradually swell and finally burst in 100% Milli-Q water (Supplementary Fig. S4). Based on this rationale, we performed two experiments to explore the bubbling mechanism (Fig. 10). The goal of first experiment was to determine whether the contents of the bubbles could be transferred back into the intracellular space by generating osmotic pressure using deionized water, Milli-Q water (Fig. 11a). Two minutes of CAP treatment was initially performed on U87MG cells without coverage of medium. After treatment, the cells were immediately immersed (< 30 s) in 6 mL of DMEM and the bubbling process was recorded over 8 min (Fig. 11b). After 8 min, the DMEM was quickly (< 1 min) removed and renewed by 6 mL of Milli-Q water in a 60 mm dish. The evolution of bubbles was recorded for 11 min after addition of Milli-Q water. Milli-Q water was theorized trigger a large osmotic pressure across the cellular membrane. Some bubbles experienced a noticeable decrease in their size over the 11 min while many bubbles fully disappeared from the site where they formed (Fig. 11c). This presented clear evidence that a given osmotic pressure could trigger the influx of the solution in the bubbles into the intracellular space.

**Figure 12:**
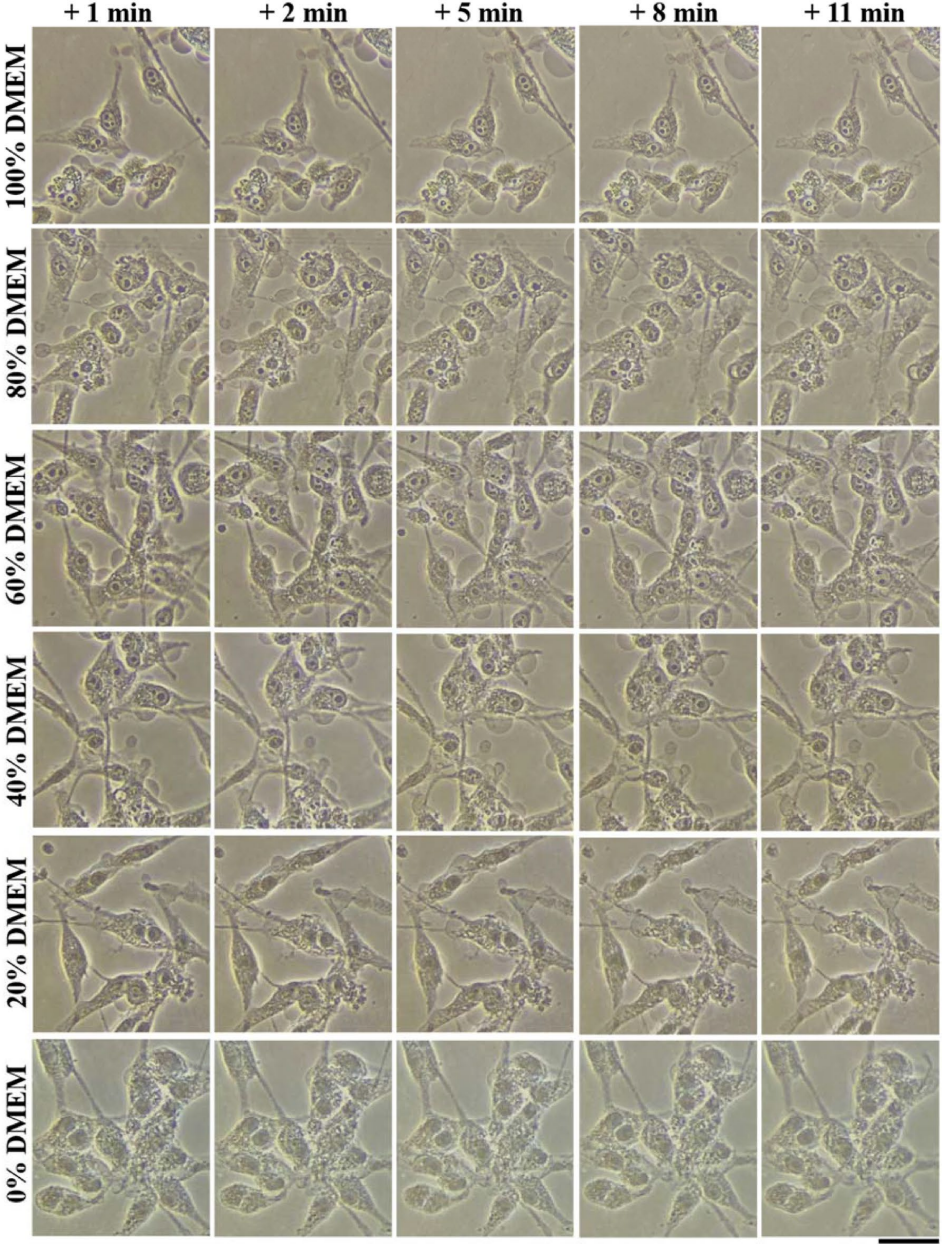
The hypotonic solutions inhibited the bubbling on the U87MG cells after the direct CAP treatment without the coverage of medium. 6 mL cell solution was cultured in a 60 mm dish with a density of 7.5 × 10^4^ cells/mL for 1 day before the treatment. In each case, the CAP treatment lasted 2 min. After that, the cells were immediately (< 30 s) immersed in 6 mL of DMEM/Milli-Q water mixed solutions. The volume ratio % (v/v) of DMEM in the solutions varied from 100 to 0%. The 0% (v/v) DMEM was 100% (v/v) Milli-Q water, a deionized water. ‘ + x min’ means the photo was taken at x min after the CAP treatment. The scale bar was 50 μm (black). In each row, the photos were taken in situ. All photos were taken by using a Nikon TS100 inverted phase-contrast microscope.

A second experiment was performed where hypotonic solutions of varying ratios of Milli-Q water and DMEM were prepared and added to dishes following CAP treatment. As the ratio of Milli-Q water gradually increased, the osmotic pressure from the extracellular environment gradually increased. As shown in Fig. 12, the bubbling on the U87MG cells drastically decreased when the volume ratio of Milli-Q water in DMEM was 80%. When the ratio reached 100% Milli-Q water, no bubbles were observed on the U87MG cells. These two experiments suggested that the bubbling was due to the intracellular pressure triggered by the physical factors in CAP.

### The physical factors that cause cell death of U87MG

**Figure 13:**
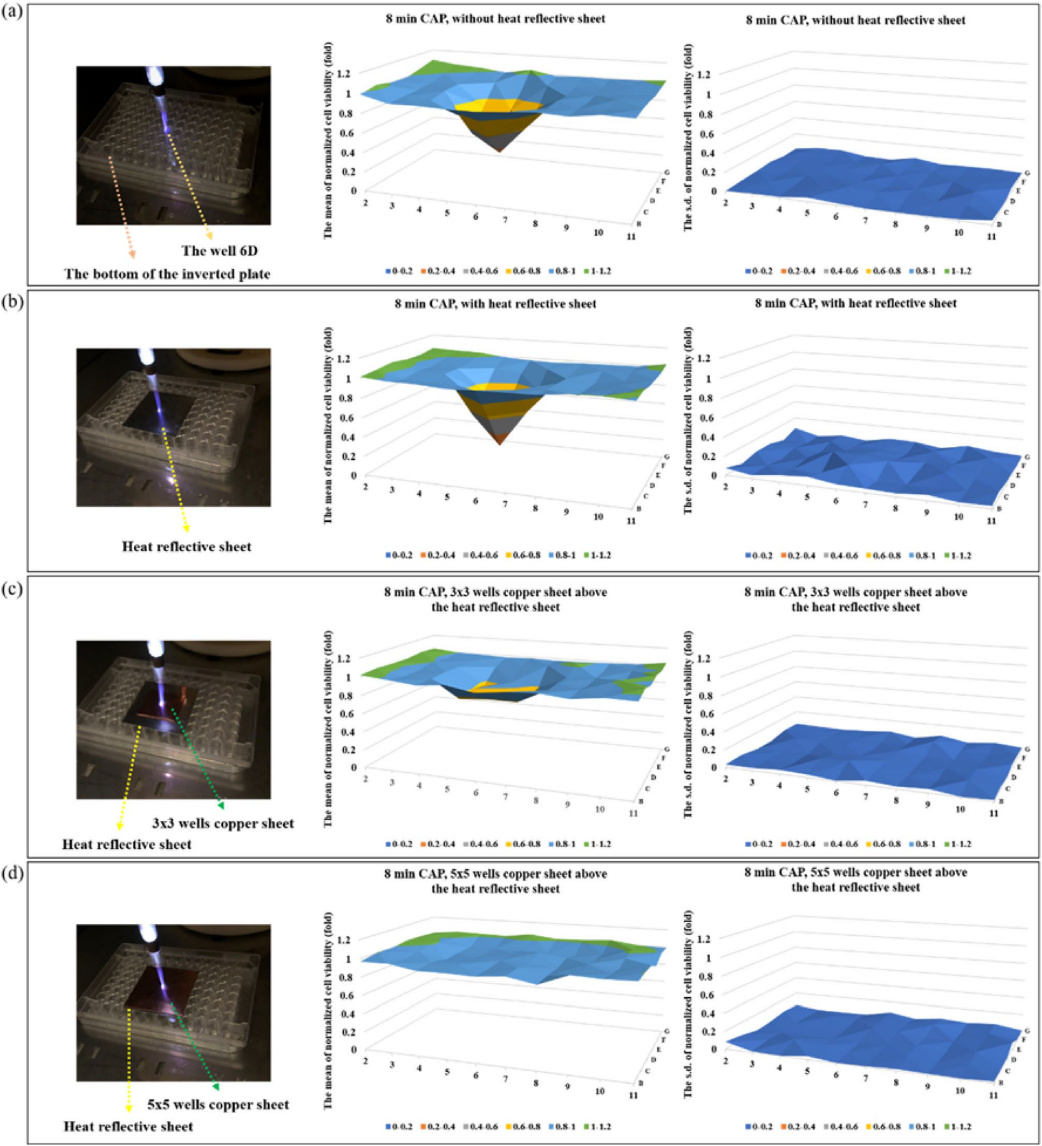
The physical anti-glioblastoma effects can be blocked by a copper sheet with an adequately large size. (**a**) Physically-based CAP treatment. (**b**) A heat-reflective sheet above the bottom of 96-well plate. (**c**) A 3 × 3 wells size copper sheet above the heat reflective sheet. (**d**) A 5 × 5 wells size copper sheet above the heat reflective sheet. The center of all these sheets was just above the well 6D. The CAP treatment was performed for 8 min in each case, targeting the well 6D. In each case, 100 μL/well of U87MG (12 × 10^4^ cell/mL) were seeded in 96-well plate and cultured for 7 h before the treatment. The cells were cultured for 2 days before the final MTT assay. The normalized cell viability was obtained by using the protocols shown in Fig. 2. In each case, the mean value and the s.d. of the normalized cell viability were presented in the left panel and the right panel, respectively. Results were presented as the mean ± s.d. of the experiments repeated 2 times. The original data were shown in Supplementary Fig. S5.

We have demonstrated that neither the heating effect nor the ultraviolet emission of CAP treatment would cause any noticeable cellular morphological change or result in the new cell death observed during physically-based CAP treatment^33^. The temperature of the tip of the CAP jet was slightly less 40 °C in this study and the ultraviolet emission was unable to penetrate the bottom of the 96-well plates or cell culture dishes. To further block the possible heating effect on the cells, we used a heat-reflective sheet (Design Engineering, 010462) to cover a 5 × 5 well area centered at well 6D on the 96-well plate in all the experiments (Fig. 13a,b). It was found that the heat-reflective sheet did not protect the cancer cells from the anti-glioblastoma effect of CAP treatment (Fig. 13b). This allowed us to solely focus on the potential role of the EM emission of CAP.

Similar to the method used in the heating effect study, the potential role of EM emission of CAP was investigated by using an ideal conductive material, a copper sheet, as an EM wave transmission blocker. The EM emission from the CAP has been demonstrated in our recent study jet and is in the range of 8–32 GHz^33^. Two different sizes of copper sheets (3 × 3 well, 5 × 5 wells) were purchased from McMaster-CARR (9709k704). The copper sheet was set between the CAP jet and the thermal reflection film above the bottom of the 96-well plate during the treatment (Fig. 13c,d). The sheet was centered at well 6D as was the CAP jet. The small copper sheet (3 × 3 wells size) strongly counteracted the anti-glioblastoma effect (Fig. 13c). A larger copper sheet (5 × 5 wells size) fully inhibited the anti-glioblastoma effect (Fig. 13d). The 'valley-shape' feature of the 2D cell viability map disappeared. Based on these results, the EM emission in the CAP jet might cause the anti-glioblastoma effect seen after physically-based CAP treatment.

## Discussion

Based on the results shown above, the physically-based anti-glioblastoma effect is due to the occurrence of a new physically-triggered cell death among the glioblastoma cells. The new cell death is a new type of necrosis characterized by the leak of water, or other cellular solution, from the aggregated cell. This observation in glioblastoma cells is highly similar to the physical effect of CAP on melanoma cells, as previously reported in our recent study^33^. Minutes after physically-based CAP treatment is performed on the cells, shrinkage of the cytoplasm occurs. This is followed by the formation and growth of bubbles from the cell membrane. The observation that bubbles continue to exist and are recognizable in Milli-Q water demonstrates that a membrane is most likely present around the surface of the bubbles. We suggest that this membrane originates from the cellular membrane. The leak of the cellular solution may cause a strong immune response if the same effect occurs in vivo. In contrast, the chemically-based CAP treatment results in apoptosis in most cases.

Generally, the cellular response to CAP treatment is believed to be mainly due to the chemical factors and is largely regarded as reactive species treatment. Based on this view, cell death after either direct CAP treatment or indirect CAP treatment is mainly due to apoptosis^37^. Even though a few studies reported the occurrence of necrosis after CAP treatment, their conclusions were just based on the flow cytometry. The direct observation of the necrosis has been rarely done. This study is one of the few cases directly showing this new type of necrotic process. The underlying mechanism triggering this new cell death remains unknown. The aggregation of the cytoplasm occurs before the bubbling, which suggests that the bubbling may be due to an increase in intracellular pressure during the aggregation of the cytoplasm. Pores may be formed on the cellular membrane, which could facilitate the bubbling. As demonstrated in our study, the electromagnetic emission from the CAP jet could be a potential factor causing this new physically-based cell death. Based on our limited understanding at the current stage, the exact connection between the EM emission and the new cell death is still unknown and is in need of further investigation. However, the strong cellular responses seen to the EM of CAP provide new hope to use physically-based rather than chemically-based CAP when the normal cell line is highly sensitive to the cytotoxicity of reactive species.

Compared to chemically-based CAP treatment, an important advantage of the physically-based CAP treatment is that the physical factors can penetrate a physical barrier on a macro scale, such as 1 mm (the thickness of the 96-well plate) in this study. The traditional chemically-based CAP treatment is largely dependent on direct contact between the bulk plasma and the aqueous layer such as medium. This direct contact allows for generation of the long-lived reactive species which are important in establishing the biological effect in vitro.

Unlike previous studies, this study aimed to examine the physically-based CAP treatment in greater detail and to build a potential correlation between in vitro and in vivo experiments. The anti-glioblastoma effect found in this study builds the foundation of using CAP as a non-invasive cancer therapy modality for subcutaneous tumor model as well as those tumor located in deeper tissues, such as glioblastomas. We propose that some of the anti-tumor effects seen during in vivo experiments involving subcutaneous tumor models may be partially due to the physically triggered necrosis. In turn, this necrosis can lead to inflammation as well as generate a stronger immune response to combat the cancer^38,39^.

## Conclusions

In summary, this study is the first to demonstrate the strong anti-glioblastoma effect achieved by implementing physically-based CAP treatment. The conventional chemically-based CAP treatment results in negative selectivity of the U87MG cells when compared with the normal human astrocyte cell line hTERT/E6/E7. This negative selectivity is determined by natural cellular responses of these two cell lines to reactive species. The physical factors of CAP could overcome the biological limitations of the chemical factors in chemically-based CAP treatment. The physical factors, mainly the EM emission, of CAP lead to a noticeable growth inhibition of U87MG cells which may be the result of a newly observed type of necrosis in U87MG cells. This necrosis is characterized by the leaking of water from the cellular membrane and the shrinkage of cytoplasm. The bubbling seen on the cell membrane may be due to physically triggered intracellular pressure, which is found to be counteracted by the osmotic pressure in deionized water. Overall the experiments in this study show a significant anti-glioblastoma effect following physically-based CAP treatment. This finding provides the foundation for further study and exploration into the exact mechanism of physically-based CAP treatment.

## Supplementary information

Supplementary figures.
