# Supplementary material for: The anti-glioblastoma effect of cold atmospheric plasma treatment: physical pathway v.s. chemical pathway
Source: Sci Rep. 2020 Jul 16;10:11788. doi: 10.1038/s41598-020-68585-z (PMC7366727; doi:10.1038/s41598-020-68585-z)
Supplement: Supplementary file 1 — Supplementary figures. [file 41598_2020_68585_MOESM1_ESM.docx]

**The Anti-glioblastoma Effect of Cold Atmospheric Plasma Treatment: Physical Way v.s. Chemical Way**

Dayun Yan^1,*,†^, Qihui Wang^1,*^, Alisa Malyavko^2,*^, Denis B.Zolotukhin^1^, Manish Adhikari^1^, Jonathan H.Sherman^3^, Michael Keidar^1,†^.

1. Department of Mechanical and Aerospace Engineering, George Washington University, Washington, DC, 20052.
2. School of Medicine and Health Science, George Washington University, Washington, DC, 20052.
3. Neurosurgery, School of Medicine and Health Science, George Washington University, Washington, DC, 20052.

*The authors are equally contributed.

†Corresponding authors: Dayun Yan, [ydy2012@gwmail.gwu.edu](mailto:ydy2012@gwmail.gwu.edu)

Michael Keidar, [keidar@gwu.edu](mailto:keidar@gwu.edu)


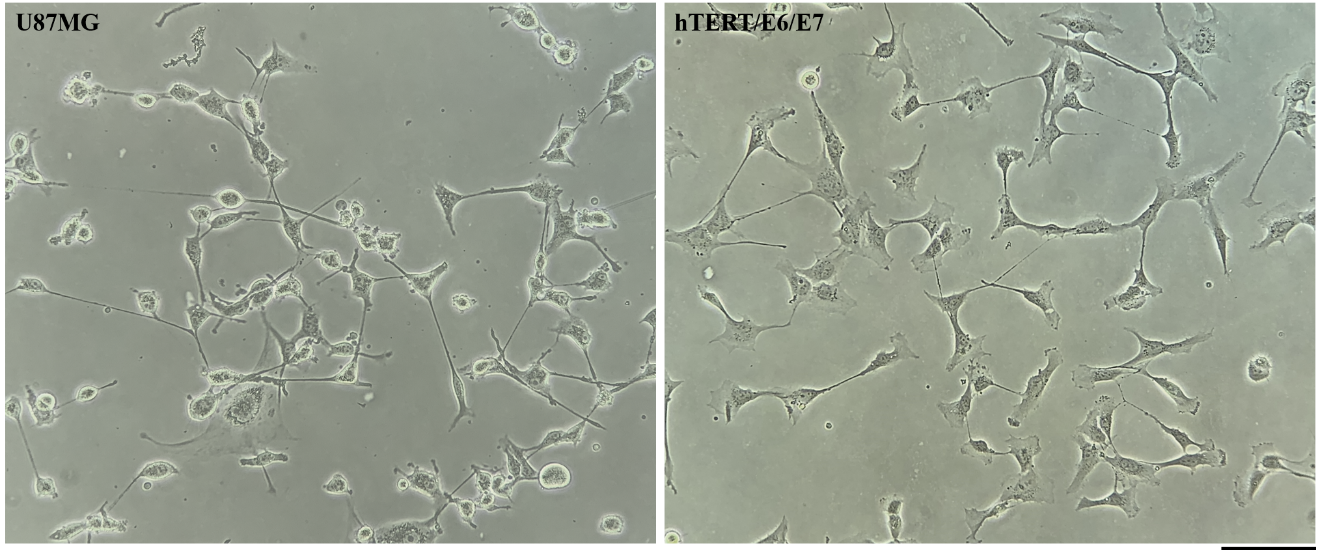


Fig. S1. The microscopic photos of the glioblastoma cell U87MG and astrocyte cell line hTERT/E6/E7 in culture. The scale bar was 100 μm (black). The photos were taken by using a Nikon TS100 inverted phase contrast microscope.

(a)

| **mean** | **1 min** |  |  |  |  |  |  |  |  |  |  |
| --- | --- | --- | --- | --- | --- | --- | --- | --- | --- | --- | --- |
|  | **2** | **3** | **4** | **5** | **6** | **7** | **8** | **9** | **10** | **11** |  |
| **B** | 0.973727247 | 1.101966709 | 1.075132428 | 1.010605965 | 0.971818352 | 0.971078561 | 0.964921422 | 0.973624441 | 1.003873314 | 0.84408787 | **B** |
| **C** | 1.11007681 | 1.050305513 | 1.001750529 | 1.004005952 | 1.089640848 | 0.951083651 | 0.885225231 | 0.926788284 | 0.965796226 | 0.880836153 | **C** |
| **D** | 1.03022264 | 1.068171653 | 1.060224746 | 1.000725743 | **0.940899665** | 1.022203798 | 0.923154815 | 0.888248341 | 0.971329714 | 0.855628115 | **D** |
| **E** | 1.125643199 | 1.0875467 | 0.987790823 | 1.057565127 | 0.983690812 | 0.96793192 | 0.897202725 | 0.920729834 | 0.968577385 | 0.880700045 | **E** |
| **F** | 1.152873628 | 1.140887691 | 1.101640261 | 1.015722812 | 1.063285362 | 0.979483641 | 0.927915454 | 0.890325713 | 0.948906351 | 0.943182077 | **F** |
| **G** | 0.989114268 | 1.152878092 | 1.133647457 | 1.065046989 | 1.049668981 | 1.033202742 | 1.025459022 | 0.994103176 | 1.007744945 | 0.879939393 | **G** |
|  | **2** | **3** | **4** | **5** | **6** | **7** | **8** | **9** | **10** | **11** |  |

(b)

| **s.d.** | **1 min** |  |  |  |  |  |  |  |  |  |  |
| --- | --- | --- | --- | --- | --- | --- | --- | --- | --- | --- | --- |
|  | **2** | **3** | **4** | **5** | **6** | **7** | **8** | **9** | **10** | **11** |  |
| **B** | 0.017899663 | 0.073058348 | 0.040295468 | 0.055965307 | 0.040914126 | 0.074450143 | 0.083993427 | 0.017122072 | 0.093511852 | 0.113170748 | **B** |
| **C** | 0.142489129 | 0.067304795 | 0.094436701 | 0.134766349 | 0.037092459 | 0.116009471 | 0.132950363 | 0.084755169 | 0.081483078 | 0.098949473 | **C** |
| **D** | 0.023750796 | 0.112695456 | 0.107402027 | 0.027434769 | **0.022094015** | 0.069238045 | 0.203543338 | 0.152946974 | 0.08477099 | 0.067812959 | **D** |
| **E** | 0.134014038 | 0.12668426 | 0.183842873 | 0.134191359 | 0.079692596 | 0.162080638 | 0.201860782 | 0.152855025 | 0.103236385 | 0.026548411 | **E** |
| **F** | 0.014269747 | 0.113591282 | 0.112560662 | 0.165727932 | 0.108870615 | 0.101282767 | 0.129701382 | 0.107283616 | 0.023468527 | 0.08482444 | **F** |
| **G** | 0.036756454 | 0.039804719 | 0.035658865 | 0.051748179 | 0.093898786 | 0.070078816 | 0.076332509 | 0.055840057 | 0.073691687 | 0.087150845 | **G** |
|  | **2** | **3** | **4** | **5** | **6** | **7** | **8** | **9** | **10** | **11** |  |

(c)

| **mean** | **2 min** |  |  |  |  |  |  |  |  |  |  |
| --- | --- | --- | --- | --- | --- | --- | --- | --- | --- | --- | --- |
|  | **2** | **3** | **4** | **5** | **6** | **7** | **8** | **9** | **10** | **11** |  |
| **B** | 0.954825085 | 1.115428007 | 1.039741862 | 1.050327588 | 0.987610327 | 0.98569601 | 1.027747966 | 1.039063715 | 1.004562097 | 0.848257158 | **B** |
| **C** | 1.096232289 | 1.101610896 | 1.090638015 | 1.028602036 | 0.975758877 | 0.999292397 | 0.93268957 | 0.977589957 | 1.051274799 | 0.944158592 | **C** |
| **D** | 1.096800924 | 1.119568513 | 1.022606732 | 0.942221442 | **0.667967948** | 0.932258587 | 0.964239982 | 0.961601307 | 1.027378563 | 0.993377341 | **D** |
| **E** | 1.063084577 | 1.123380846 | 1.084702688 | 0.970689951 | 0.961663172 | 0.98250472 | 0.933445977 | 0.944171175 | 0.9979722 | 0.977667399 | **E** |
| **F** | 1.030709659 | 1.09706487 | 1.055608372 | 1.012794045 | 1.028611273 | 0.994301503 | 0.940007075 | 0.982042621 | 0.978487361 | 0.944680822 | **F** |
| **G** | 0.98200976 | 1.116223342 | 1.049865575 | 1.045056765 | 1.011901899 | 0.995035556 | 0.948210404 | 0.945711934 | 1.027839893 | 0.815703383 | **G** |
|  | **2** | **3** | **4** | **5** | **6** | **7** | **8** | **9** | **10** | **11** |  |

(d)

| **s.d.** | **2 min** |  |  |  |  |  |  |  |  |  |  |
| --- | --- | --- | --- | --- | --- | --- | --- | --- | --- | --- | --- |
|  | **2** | **3** | **4** | **5** | **6** | **7** | **8** | **9** | **10** | **11** |  |
| **B** | 0.013134123 | 0.087327628 | 0.02550683 | 0.066741876 | 0.02925467 | 0.023731398 | 0.074566496 | 0.056327749 | 0.048215106 | 0.075218457 | **B** |
| **C** | 0.016980372 | 0.053241646 | 0.044330281 | 0.083803265 | 0.058856465 | 0.046994239 | 0.059302757 | 0.082133538 | 0.059518634 | 0.032650096 | **C** |
| **D** | 0.013234372 | 0.053149897 | 0.072445581 | 0.072607639 | **0.094306849** | 0.076994885 | 0.093242527 | 0.11898376 | 0.082443328 | 0.008834101 | **D** |
| **E** | 0.052053941 | 0.028583486 | 0.041335421 | 0.039835946 | 0.065607801 | 0.091992914 | 0.102422629 | 0.120433899 | 0.052822699 | 0.017757493 | **E** |
| **F** | 0.066820072 | 0.084865509 | 0.072497161 | 0.057914768 | 0.0887026 | 0.100639865 | 0.077048345 | 0.073374222 | 0.067246648 | 0.033388642 | **F** |
| **G** | 0.030818654 | 0.068502654 | 0.050704236 | 0.057365708 | 0.039050817 | 0.061240111 | 0.073926027 | 0.094609012 | 0.082043537 | 0.093191714 | **G** |
|  | **2** | **3** | **4** | **5** | **6** | **7** | **8** | **9** | **10** | **11** |  |

(e)

| **mean** | **4 min** |  |  |  |  |  |  |  |  |  |  |
| --- | --- | --- | --- | --- | --- | --- | --- | --- | --- | --- | --- |
|  | **2** | **3** | **4** | **5** | **6** | **7** | **8** | **9** | **10** | **11** |  |
| **B** | 0.921688042 | 1.058004654 | 1.074105273 | 1.007573441 | 1.026711957 | 0.985910133 | 0.984440252 | 1.001650368 | 1.018063859 | 0.950012434 | **B** |
| **C** | 0.988276739 | 1.066499485 | 1.05012723 | 1.010971083 | 0.793180312 | 1.046429849 | 0.9627702 | 0.946663438 | 1.033077372 | 1.005698634 | **C** |
| **D** | 1.015479087 | 1.089232731 | 1.057828782 | 0.704854561 | **0.2394715** | 0.811698303 | 0.987067458 | 0.943129747 | 1.005769875 | 0.99236488 | **D** |
| **E** | 0.99167468 | 1.170592018 | 1.085355942 | 0.886759457 | 0.887272886 | 0.903762049 | 0.990202641 | 1.001492149 | 1.059798484 | 1.029855156 | **E** |
| **F** | 1.022035781 | 1.060405926 | 1.080493071 | 1.077194327 | 1.036249956 | 1.008768143 | 1.007495089 | 1.01112712 | 1.024505363 | 0.942554411 | **F** |
| **G** | 0.944619525 | 1.043388485 | 1.035676298 | 1.054510234 | 1.056421108 | 1.044365096 | 0.978148489 | 1.041184059 | 1.012486312 | 0.851259976 | **G** |
|  | **2** | **3** | **4** | **5** | **6** | **7** | **8** | **9** | **10** | **11** |  |

(f)

| **s.d.** | **4 min** |  |  |  |  |  |  |  |  |  |  |
| --- | --- | --- | --- | --- | --- | --- | --- | --- | --- | --- | --- |
|  | **2** | **3** | **4** | **5** | **6** | **7** | **8** | **9** | **10** | **11** |  |
| **B** | 0.004370242 | 0.076890017 | 0.080179308 | 0.072309532 | 0.069954862 | 0.061120868 | 0.071736479 | 0.051890317 | 0.062601442 | 0.107561319 | **B** |
| **C** | 0.049285862 | 0.042936167 | 0.113020033 | 0.037223609 | 0.061185386 | 0.041124003 | 0.106393152 | 0.158345153 | 0.029377928 | 0.093921785 | **C** |
| **D** | 0.110906955 | 0.078038411 | 0.089043072 | 0.061816789 | **0.084902072** | 0.013432471 | 0.108062436 | 0.042251027 | 0.031986641 | 0.07660487 | **D** |
| **E** | 0.059879067 | 0.109451242 | 0.019662516 | 0.020418493 | 0.046938794 | 0.017571962 | 0.047156814 | 0.052322162 | 0.035695069 | 0.09882689 | **E** |
| **F** | 0.049279085 | 0.113117728 | 0.05518897 | 0.053815543 | 0.081342886 | 0.071160456 | 0.120834911 | 0.097881639 | 0.034790117 | 0.056972108 | **F** |
| **G** | 0.030953667 | 0.153560254 | 0.049762265 | 0.037901893 | 0.036786021 | 0.006947993 | 0.04512374 | 0.060142211 | 0.037408682 | 0.055193674 | **G** |
|  | **2** | **3** | **4** | **5** | **6** | **7** | **8** | **9** | **10** | **11** |  |

(g)

| **mean** | **8 min** |  |  |  |  |  |  |  |  |  |  |
| --- | --- | --- | --- | --- | --- | --- | --- | --- | --- | --- | --- |
|  | **2** | **3** | **4** | **5** | **6** | **7** | **8** | **9** | **10** | **11** |  |
| **B** | 0.971038873 | 1.091964775 | 1.085249392 | 1.028474552 | 1.020679711 | 0.971206696 | 0.963766718 | 0.968887325 | 1.003723114 | 1.011424069 | **B** |
| **C** | 1.029303721 | 1.053330565 | 0.946010812 | 1.005594322 | 0.743005704 | 0.985082033 | 0.852809125 | 0.91873457 | 0.985731913 | 0.96265291 | **C** |
| **D** | 1.025019257 | 1.062425166 | 0.956849618 | 0.574615957 | **0.120406216** | 0.791074384 | 0.906155275 | 0.86507841 | 1.01901022 | 0.960344335 | **D** |
| **E** | 0.940465827 | 1.114561139 | 0.939512014 | 0.834855344 | 0.742273676 | 0.885740128 | 0.903974058 | 0.886890458 | 1.078126857 | 0.950305867 | **E** |
| **F** | 0.989260171 | 1.106314418 | 0.971905147 | 1.02243891 | 1.00813412 | 0.929423946 | 0.950500249 | 0.880436514 | 1.040766264 | 0.968321136 | **F** |
| **G** | 1.024793665 | 1.12453348 | 1.105586107 | 1.075814921 | 1.041449722 | 1.008298417 | 0.974844536 | 1.008668148 | 1.013419024 | 1.034005628 | **G** |
|  | **2** | **3** | **4** | **5** | **6** | **7** | **8** | **9** | **10** | **11** |  |

(h)

| **s.d.** | **8 min** |  |  |  |  |  |  |  |  |  |  |
| --- | --- | --- | --- | --- | --- | --- | --- | --- | --- | --- | --- |
|  | **2** | **3** | **4** | **5** | **6** | **7** | **8** | **9** | **10** | **11** |  |
| **B** | 0.072860679 | 0.049996722 | 0.05140715 | 0.011675844 | 0.070501508 | 0.086928572 | 0.028615274 | 0.015637391 | 0.004946598 | 0.034256505 | **B** |
| **C** | 0.038147001 | 0.035798729 | 0.038429247 | 0.013329137 | 0.119993689 | 0.011089552 | 0.055511312 | 0.016688424 | 0.012008621 | 0.081129217 | **C** |
| **D** | 0.101363286 | 0.024865072 | 0.007804191 | 0.124689262 | **0.09611857** | 0.142083059 | 0.031694725 | 0.032423672 | 0.001708742 | 0.05053019 | **D** |
| **E** | 0.123020318 | 0.102172322 | 0.047619936 | 0.138987838 | 0.17249679 | 0.054158923 | 0.065372772 | 0.02834595 | 0.039422282 | 0.084985401 | **E** |
| **F** | 0.126547057 | 0.033527428 | 0.041962887 | 0.040975148 | 0.116349308 | 0.037029529 | 0.067348131 | 0.054306815 | 0.057333523 | 0.091950645 | **F** |
| **G** | 0.06462593 | 0.038128226 | 0.028821165 | 0.015119788 | 0.039216194 | 0.009504969 | 0.062663051 | 0.064543418 | 0.022482852 | 0.061854493 | **G** |
|  | **2** | **3** | **4** | **5** | **6** | **7** | **8** | **9** | **10** | **11** |  |

Fig. S2. The original data to draw the 2D cell viability maps in Fig. 4. (a) mean value of 1 min treatment; (b) s.d. of 1 min treatment; (c) mean value of 2 min treatment; (d) s.d. of 2 min treatment; (e) mean value of 3 min treatment; (f) s.d. of 3 min treatment; (g) mean value of 4 min treatment; (h) s.d. of 4 min treatment. Results are presented as the mean ± s.d. of the experiments repeated for 4 times.


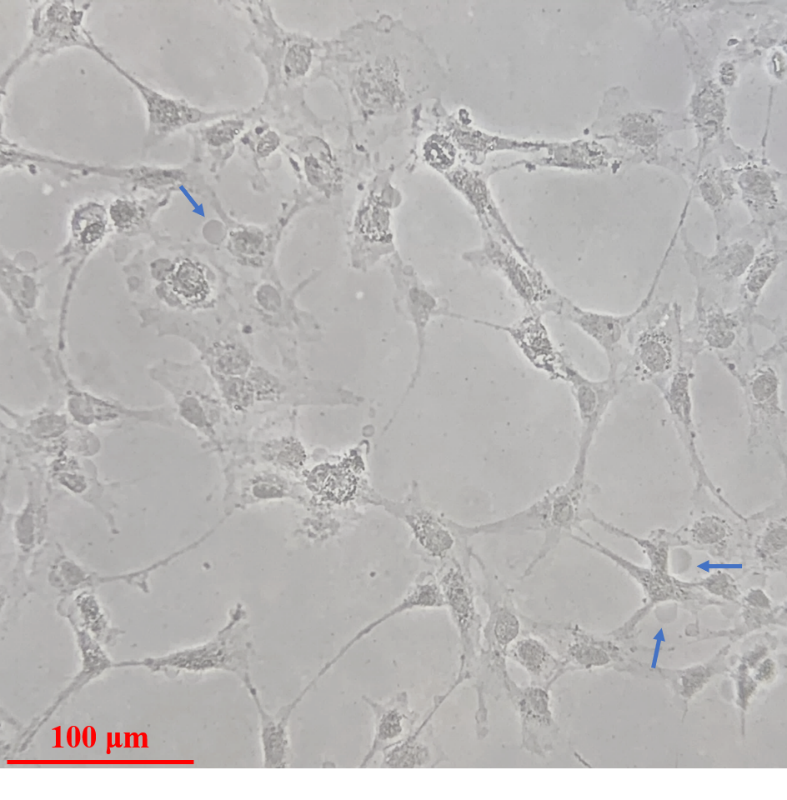


Fig. S3. Image representing the morphological change of the CAP-treated hTERT/E6/E7 cells. The photo was taken after a 4 min of CAP treatment. The photo was taken at 11 min after this CAP treatment. The few distinguishable bubbles are marked by blue arrows.


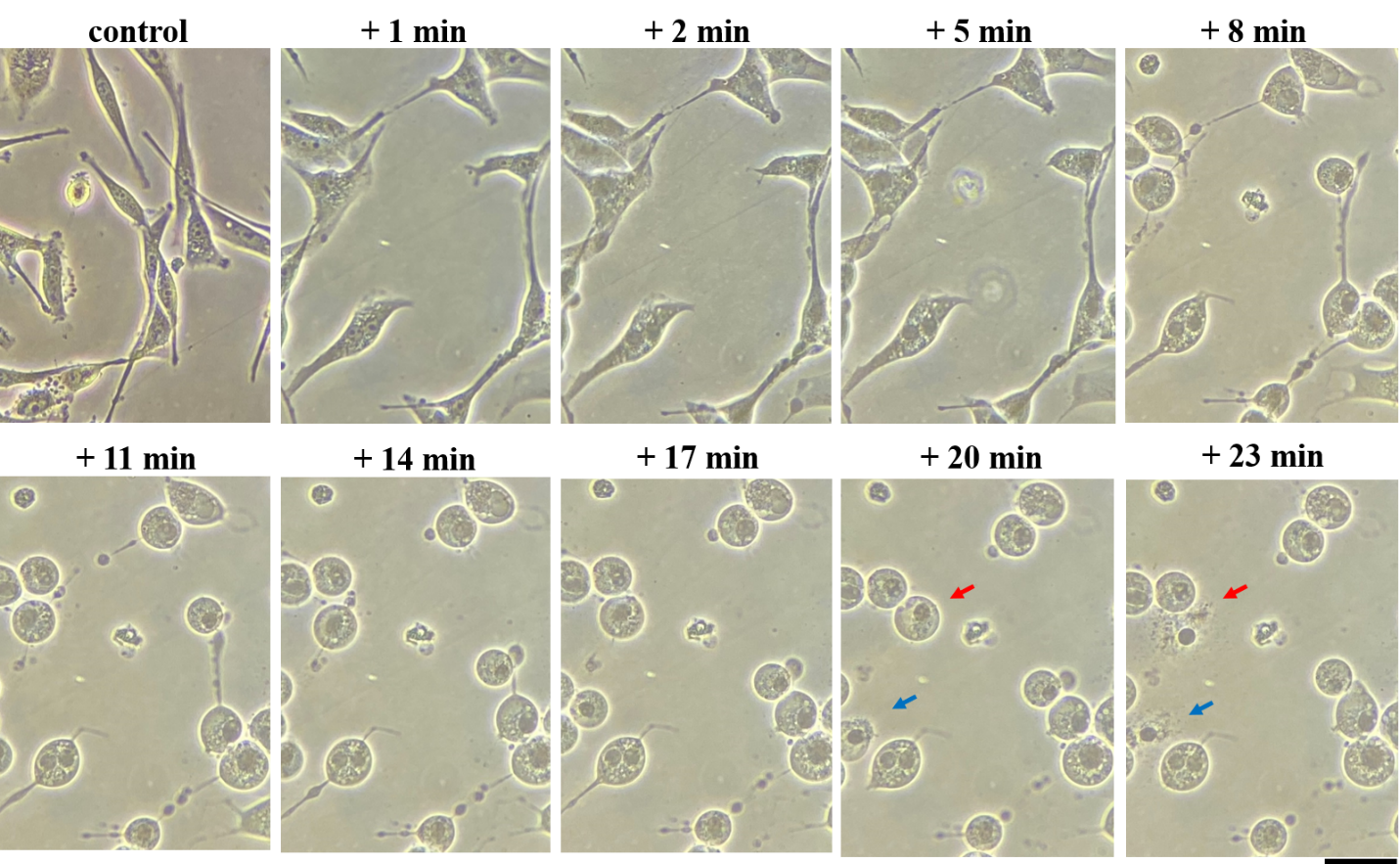


## Fig. S4. Milli-Q water swells the U87MG cells without experiencing the CAP treatment. 6 mL cell solution was cultured in a 60 mm dish with a density of 7.5 x 10^4^ cells/mL for one day before the treatment. After removing medium, U87MG cells were immediately (<30 s) immersed in 6 mL of Milli-Q water. ‘+ x min’ means the photo was taken at x min after the treatment. The scale bar was 50 μm (black). The photos in the experimental groups were taken in situ. Red and blue arrows mark two specific cells, which experienced clear burst. All photos were taken by using a Nikon TS100 inverted phase-contrast microscope.

(a)

| **mean** | **8 min** |  |  | **no thermal** | **reflection film** |  |  |  |  |  |  |
| --- | --- | --- | --- | --- | --- | --- | --- | --- | --- | --- | --- |
|  | **2** | **3** | **4** | **5** | **6** | **7** | **8** | **9** | **10** | **11** |  |
| **B** | 0.98881246 | 0.918835489 | 0.922107133 | 0.911773803 | 0.911697877 | 0.931289523 | 0.951855123 | 0.938743408 | 0.976763192 | 0.970198956 | **B** |
| **C** | 1.029125748 | 0.926791012 | 0.908666578 | 0.778527569 | 0.562761835 | 0.797866302 | 0.918578385 | 0.93521047 | 0.956783536 | 0.986278611 | **C** |
| **D** | 0.99398122 | 0.935142922 | 0.944245197 | 0.515926204 | **0.341219931** | 0.643906805 | 0.92751205 | 0.922275743 | 0.986253476 | 0.987008029 | **D** |
| **E** | 0.992100862 | 0.954485844 | 0.954076889 | 0.738467196 | 0.719870448 | 0.766570984 | 0.909404374 | 0.940067147 | 0.962850323 | 0.976860065 | **E** |
| **F** | 1.036583821 | 0.997092634 | 0.955784448 | 0.933081387 | 0.850642002 | 0.93719189 | 0.939561319 | 0.956526433 | 0.984309759 | 0.995444244 | **F** |
| **G** | 1.085839152 | 1.046516575 | 1.02944202 | 0.990203747 | 1.039863845 | 0.986586505 | 0.996025998 | 0.991759454 | 0.99799485 | 1.012413548 | **G** |
|  | **2** | **3** | **4** | **5** | **6** | **7** | **8** | **9** | **10** | **11** |  |

(b)

| **s.d.** | **8 min** |  |  | **no thermal** | **reflection film** |  |  |  |  |  |  |
| --- | --- | --- | --- | --- | --- | --- | --- | --- | --- | --- | --- |
|  | **2** | **3** | **4** | **5** | **6** | **7** | **8** | **9** | **10** | **11** |  |
| **B** | 0.001874508 | 0.009442205 | 0.011879013 | 0.041088312 | 0.020112919 | 0.00435035 | 0.003865783 | 0.006191034 | 0.014049493 | 0.044415492 | **B** |
| **C** | 0.012976469 | 0.033667025 | 0.017912817 | 0.059243045 | 0.08381888 | 0.059463721 | 0.029843428 | 0.038757136 | 0.037160826 | 0.020490111 | **C** |
| **D** | 0.020030985 | 0.011421631 | 0.034024699 | 0.042933953 | **0.037502932** | 0.01284985 | 0.023696323 | 0.004576951 | 0.067120012 | 0.007669863 | **D** |
| **E** | 0.035211023 | 0.025802642 | 0.028467794 | 0.023457138 | 0.007591374 | 0.033986674 | 0.016869417 | 0.037846552 | 0.047362309 | 0.037983551 | **E** |
| **F** | 0.008916166 | 0.011457178 | 0.017769895 | 0.033659357 | 0.046454163 | 0.012697557 | 0.011521339 | 0.002124807 | 0.012840479 | 0.021687275 | **F** |
| **G** | 0.015332351 | 0.052161659 | 0.053072978 | 0.008015688 | 0.00103401 | 0.048809772 | 0.006705224 | 0.012302592 | 0.014354855 | 0.002310944 | **G** |
|  | **2** | **3** | **4** | **5** | **6** | **7** | **8** | **9** | **10** | **11** |  |

(c)

| **mean** | **8 min** |  |  | **with thermal** | **reflection film** | **5x5 wells** |  |  |  |  |  |
| --- | --- | --- | --- | --- | --- | --- | --- | --- | --- | --- | --- |
|  | **2** | **3** | **4** | **5** | **6** | **7** | **8** | **9** | **10** | **11** |  |
| **B** | 1.012312953 | 0.987401271 | 0.983846984 | 0.961044761 | 0.968884547 | 0.973331365 | 0.974381695 | 1.005598877 | 1.009719539 | 0.992414464 | **B** |
| **C** | 1.028399341 | 0.945180995 | 0.932647135 | 0.924807348 | 0.64097889 | 0.931439005 | 0.953513535 | 0.926260975 | 0.964428932 | 1.014089217 | **C** |
| **D** | 1.047892774 | 0.975510925 | 0.964425412 | 0.556797912 | **0.251443703** | 0.642674495 | 0.949715511 | 0.916793927 | 0.967581682 | 0.989012697 | **D** |
| **E** | 1.023228349 | 0.963780138 | 0.959820795 | 0.728226681 | 0.675583762 | 0.791218621 | 0.933374828 | 0.907253257 | 0.949548913 | 0.990226106 | **E** |
| **F** | 1.019826583 | 0.989014457 | 0.973401467 | 0.927714602 | 0.880973887 | 0.952382546 | 0.947523634 | 0.975186529 | 0.980922135 | 0.994676443 | **F** |
| **G** | 1.047328159 | 1.036160227 | 1.028150325 | 0.99184457 | 0.975423228 | 0.966285855 | 0.978826754 | 0.980597738 | 0.98456412 | 1.026538899 | **G** |
|  | **2** | **3** | **4** | **5** | **6** | **7** | **8** | **9** | **10** | **11** |  |

(d)

| **s.d.** | **8 min** |  |  | **with thermal** | **reflection film** | **5x5 wells** |  |  |  |  |  |
| --- | --- | --- | --- | --- | --- | --- | --- | --- | --- | --- | --- |
|  | **2** | **3** | **4** | **5** | **6** | **7** | **8** | **9** | **10** | **11** |  |
| **B** | 0.077019395 | 0.03355605 | 0.069694066 | 0.090960752 | 0.050592212 | 0.042473374 | 0.033667631 | 0.057233124 | 0.023954309 | 0.028296413 | **B** |
| **C** | 0.019498135 | 0.038501798 | 0.033586859 | 0.006781681 | 0.144643066 | 0.012043806 | 0.008193766 | 0.011744849 | 0.023630467 | 0.050716235 | **C** |
| **D** | 0.061473601 | 0.006449427 | 0.056567074 | 0.159377932 | **0.155653056** | 0.120284064 | 0.01730842 | 0.064239588 | 0.033579393 | 0.021795402 | **D** |
| **E** | 0.061582694 | 0.041013815 | 0.011623314 | 0.072498522 | 0.086553948 | 0.040147193 | 0.034615972 | 0.048916956 | 0.028208176 | 0.018021157 | **E** |
| **F** | 0.011490878 | 0.005327099 | 0.058280604 | 0.030271379 | 0.026393018 | 0.002556135 | 0.061945757 | 0.002242247 | 0.057935907 | 0.006796612 | **F** |
| **G** | 0.075082703 | 0.002457944 | 0.040542606 | 0.007499395 | 0.05747963 | 0.051877785 | 0.009080489 | 0.066627581 | 0.030144868 | 0.014808846 | **G** |
|  | **2** | **3** | **4** | **5** | **6** | **7** | **8** | **9** | **10** | **11** |  |

(e)

| **mean** | **8 min** |  |  | **with thermal** | **reflection film** | **5x5 wells** |  | **copper sheet** | **3x3 wells** |  |  |
| --- | --- | --- | --- | --- | --- | --- | --- | --- | --- | --- | --- |
|  | **2** | **3** | **4** | **5** | **6** | **7** | **8** | **9** | **10** | **11** |  |
| **B** | 1.018997677 | 0.988863739 | 0.956448624 | 0.984957552 | 0.929251497 | 0.963909681 | 0.977107602 | 0.944692488 | 0.983080935 | 0.975024789 | **B** |
| **C** | 1.034453804 | 0.96040022 | 0.955929747 | 0.762720058 | 0.783775762 | 0.780220892 | 0.932455579 | 0.960896128 | 0.985766141 | 1.024704268 | **C** |
| **D** | 1.023315727 | 0.9622617 | 0.96805964 | 0.798072513 | **0.783386869** | 0.76296436 | 0.935942071 | 0.93073922 | 1.010339138 | 0.98337848 | **D** |
| **E** | 1.023315727 | 1.001573585 | 0.963078123 | 0.770860249 | 0.823507342 | 0.792411861 | 0.925253961 | 0.944471155 | 0.937506006 | 1.022598486 | **E** |
| **F** | 1.044981656 | 0.975604742 | 0.970432165 | 0.918685539 | 0.945005169 | 0.983157147 | 1.023644074 | 0.949277279 | 0.982981754 | 1.001245237 | **F** |
| **G** | 1.034987818 | 1.001542782 | 0.991732171 | 0.977504328 | 0.984789991 | 0.9742162 | 0.975192879 | 0.984705946 | 0.99175514 | 1.031882917 | **G** |
|  | **2** | **3** | **4** | **5** | **6** | **7** | **8** | **9** | **10** | **11** |  |

(f)

| **s.d.** | **8 min** |  |  | **with thermal** | **reflection film** | **5x5 wells** |  | **copper sheet** | **3x3 wells** |  |  |
| --- | --- | --- | --- | --- | --- | --- | --- | --- | --- | --- | --- |
|  | **2** | **3** | **4** | **5** | **6** | **7** | **8** | **9** | **10** | **11** |  |
| **B** | 0.031589735 | 0.029669362 | 0.04651061 | 0.033165122 | 0.006257117 | 0.046316456 | 0.025580303 | 0.042421551 | 0.003582014 | 0.014975125 | **B** |
| **C** | 0.034804727 | 0.008793185 | 0.009957868 | 0.102096671 | 0.115302135 | 0.052273498 | 0.010702989 | 0.00086287 | 0.057094861 | 0.026627118 | **C** |
| **D** | 0.000409834 | 0.001003134 | 0.005124917 | 0.048518856 | **0.056750865** | 0.044440874 | 0.017509964 | 0.035225279 | 0.051085429 | 0.002211619 | **D** |
| **E** | 0.000409834 | 0.065553268 | 0.043349508 | 0.049392923 | 0.01613047 | 0.052942333 | 0.007767536 | 0.011662441 | 0.021512649 | 0.040824997 | **E** |
| **F** | 0.041407459 | 0.035991566 | 0.025051971 | 0.025342587 | 0.008835757 | 0.028547491 | 0.0985566 | 0.013777717 | 0.005513225 | 0.033413166 | **F** |
| **G** | 0.037631411 | 0.027619533 | 0.019398362 | 0.033305146 | 0.056145258 | 0.008954615 | 0.067646149 | 0.014834621 | 0.04629505 | 0.018740095 | **G** |
|  | **2** | **3** | **4** | **5** | **6** | **7** | **8** | **9** | **10** | **11** |  |

(g)

| **mean** | **8 min** |  |  | **with thermal** | **reflection film** | **5x5 wells** |  | **copper sheet** | **5x5 wells** |  |  |
| --- | --- | --- | --- | --- | --- | --- | --- | --- | --- | --- | --- |
|  | **2** | **3** | **4** | **5** | **6** | **7** | **8** | **9** | **10** | **11** |  |
| **B** | 0.965992614 | 0.9680643 | 0.916883384 | 0.917034969 | 0.907765409 | 0.934603937 | 0.878569702 | 0.998801146 | 0.985711585 | 0.992919559 | **B** |
| **C** | 1.002469563 | 0.948057309 | 0.936380028 | 0.943729487 | 0.907469815 | 0.947915825 | 0.89423877 | 0.934310868 | 0.96225093 | 1.001815207 | **C** |
| **D** | 0.995931095 | 0.964461575 | 0.895706641 | 0.919384573 | **0.933206808** | 0.926299478 | 0.899314416 | 0.967768705 | 0.938287504 | 0.994167629 | **D** |
| **E** | 1.022254226 | 1.011451103 | 0.910046797 | 0.911590461 | 0.943143348 | 0.97674772 | 0.940637109 | 0.944020031 | 0.980936584 | 0.924005466 | **E** |
| **F** | 1.016440857 | 0.992767975 | 0.917626157 | 0.919609426 | 0.914907691 | 0.903435062 | 0.896598474 | 0.939533048 | 1.017923878 | 0.997108422 | **F** |
| **G** | 1.000559562 | 1.030712795 | 1.024022742 | 1.016152837 | 1.053076965 | 1.016670759 | 1.025058585 | 1.043880673 | 1.001969317 | 0.989680646 | **G** |
|  | **2** | **3** | **4** | **5** | **6** | **7** | **8** | **9** | **10** | **11** |  |

(h)

| **s.d.** | **8 min** |  |  | **with thermal** | **reflection film** | **5x5 wells** |  | **copper sheet** | **5x5 wells** |  |  |
| --- | --- | --- | --- | --- | --- | --- | --- | --- | --- | --- | --- |
|  | **2** | **3** | **4** | **5** | **6** | **7** | **8** | **9** | **10** | **11** |  |
| **B** | 0.083111728 | 0.011314097 | 0.001317001 | 0.034802653 | 0.002978422 | 0.006893633 | 0.017515993 | 0.009034552 | 0.005079303 | 0.017352372 | **B** |
| **C** | 0.052320082 | 0.031536245 | 0.076716884 | 0.047600943 | 0.007836886 | 0.009852765 | 0.029596874 | 0.001009725 | 0.019781254 | 0.002899803 | **C** |
| **D** | 0.007501043 | 0.014536895 | 0.008798754 | 0.009013127 | **0.006790017** | 0.029189553 | 0.043013287 | 0.000498788 | 0.044063727 | 0.002481769 | **D** |
| **E** | 0.022260888 | 0.024844838 | 0.016602066 | 0.012546772 | 0.063407658 | 0.058945042 | 0.0182742 | 0.02097892 | 0.03991553 | 0.078027419 | **E** |
| **F** | 0.053356239 | 0.016133281 | 0.05643327 | 0.04052294 | 0.073383328 | 0.063396945 | 0.07868201 | 0.010474459 | 0.038157635 | 0.001677141 | **F** |
| **G** | 0.038385591 | 0.006139756 | 0.005193631 | 0.00801555 | 0.067077057 | 0.015590906 | 0.052406543 | 0.033276986 | 0.055104121 | 0.008022692 | **G** |
|  | **2** | **3** | **4** | **5** | **6** | **7** | **8** | **9** | **10** | **11** |  |

(i)

| **mean** | **8 min** |  |  | **with thermal** | **reflection film** | **5x5 wells** |  | **copper sheet** | **10x6 wells** |  |  |
| --- | --- | --- | --- | --- | --- | --- | --- | --- | --- | --- | --- |
|  | **2** | **3** | **4** | **5** | **6** | **7** | **8** | **9** | **10** | **11** |  |
| **B** | 0.952230239 | 0.989633691 | 1.00078278 | 0.955330167 | 0.955596637 | 0.957810112 | 0.980403183 | 1.001372567 | 0.980403183 | 0.980255736 | **B** |
| **C** | 1.052499207 | 0.976290668 | 0.959938315 | 0.938045171 | 0.941315641 | 0.943291069 | 0.9624378 | 0.956577246 | 0.97285321 | 0.977827311 | **C** |
| **D** | 0.984753745 | 0.937883512 | 0.940958572 | 0.929262331 | **0.971382298** | 0.930271363 | 0.951135936 | 0.943676562 | 0.985691718 | 0.961199605 | **D** |
| **E** | 1.03702798 | 0.99359876 | 0.945966425 | 0.949951033 | 0.973181856 | 0.972048473 | 0.953740232 | 1.002234152 | 0.948389522 | 0.987015183 | **E** |
| **F** | 0.995273967 | 0.982497635 | 0.957781688 | 0.937673889 | 0.952283531 | 0.941953392 | 0.964946168 | 0.973419902 | 0.963118186 | 0.958195605 | **F** |
| **G** | 1.060001217 | 1.058654655 | 1.041811999 | 1.031725234 | 1.014244827 | 1.015134836 | 1.016325066 | 1.010693674 | 0.994341322 | 1.020332771 | **G** |
|  | **2** | **3** | **4** | **5** | **6** | **7** | **8** | **9** | **10** | **11** |  |

(j)

| **s.d.** | **8 min** |  |  | **with thermal** | **reflection film** | **5x5 wells** |  | **copper sheet** | **10x6 wells** |  |  |
| --- | --- | --- | --- | --- | --- | --- | --- | --- | --- | --- | --- |
|  | **2** | **3** | **4** | **5** | **6** | **7** | **8** | **9** | **10** | **11** |  |
| **B** | 0.078379832 | 0.052071332 | 0.081626026 | 0.055261954 | 0.038404422 | 0.020853496 | 0.042464298 | 0.006628854 | 0.042464298 | 0.061213591 | **B** |
| **C** | 0.024473419 | 0.114203018 | 0.065225735 | 0.06528603 | 0.075081487 | 0.053747037 | 0.104952731 | 0.032897461 | 0.049021401 | 0.030116042 | **C** |
| **D** | 0.025490898 | 0.094355851 | 0.069406202 | 0.083887098 | **0.057281842** | 0.057739082 | 0.009691348 | 0.038781268 | 3.62772E-05 | 0.032540715 | **D** |
| **E** | 0.006713971 | 0.000918095 | 0.054083685 | 0.075229712 | 0.075337741 | 0.015138014 | 0.061630634 | 0.000769869 | 0.060957338 | 0.010452574 | **E** |
| **F** | 0.069209939 | 0.02302161 | 0.041494551 | 0.069931273 | 0.059570548 | 0.053578713 | 0.049903218 | 0.079121264 | 0.052488375 | 0.005887726 | **F** |
| **G** | 0.08452492 | 0.010083264 | 0.036140539 | 0.046107131 | 0.021386102 | 0.006653676 | 0.012263942 | 0.038768704 | 0.010208579 | 0.071687067 | **G** |
|  | **2** | **3** | **4** | **5** | **6** | **7** | **8** | **9** | **10** | **11** |  |

Fig. S5. The original data to draw the 2D cell viability maps in Fig. 12. The data used to draw the 2D cell viability maps in Fig. 12a (left and right panel), Fig. 12b (left and right panel), Fig. 12c (left and right panel), Fig. 12d (left and right panel), and Fig. 12e (left and right panel) were shown in (a), (b), (c), (d), (e), (f), (g), (h), (i), and (j), respectively. Results are presented as the mean ± s.d. of the experiments repeated for 2 times.
